# Attitude Uncertainty Analysis of a Three-Vehicle Constrained Formation

**DOI:** 10.3390/s22103879

**Published:** 2022-05-20

**Authors:** Pedro Cruz, Pedro Batista

**Affiliations:** 1 Instituto Superior Técnico, Universidade de Lisboa, 1049-001 Lisboa, Portugal; pbatista@isr.tecnico.ulisboa.pt; 2 Laboratory for Robotics and Engineering Systems, Institute for Systems and Robotics, 1049-001 Lisboa, Portugal

**Keywords:** uncertainty analysis, attitude determination, formation of vehicles, constrained formation

## Abstract

The uncertainty analysis of attitude estimates enables the comparison between different methods, and, thus, it is important for practical applications. This work studies the uncertainty for the attitude determination of a three-vehicle constrained formation. Moreover, the existing solution is improved by including the uncertainty results in a weighted orthogonal Procrustes problem. In the formation considered herein, the vehicles measure inertial references and relative line-of-sight vectors. Nonetheless, the line of sight between two elements of the formation is restricted. The uncertainty analysis uses perturbation theory and, consequently, considers a small first-order perturbation in the measurements. The covariance matrices are obtained for all relative and inertial attitude candidates from the linearization of the solution using a first-order Taylor expansion. Then, the uncertainty is completed by considering the covariance for the weighted orthogonal Procrustes problem, from the literature, and the definition of covariance for the remaining attitudes. The uncertainty characterization is valid for configurations with a unique solution. Finally, the theoretical results are validated by applying Monte Carlo simulations, which show that the predicted errors are statistically consistent with the numerical implementation of the solution with noise. Furthermore, the theoretical uncertainty predicts the accuracy changes near special configurations where there is loss of information.

## 1. Introduction

Potential advantages in autonomy, reliability, and accuracy have driven the design of autonomous vehicle formations [1]. In the context of space missions, there has been extensive work published regarding the guidance, navigation, and control of spacecraft formation flying missions; see [2,3].

Constrained formations must satisfy a set of constraints, which are imposed by design or by the environment. Specifically, in the context of space missions, such formations could be applied to large synthetic aperture telescopes or long baseline interferometers, close to or far from the Earth, or even to sample spatially disperse phenomena such as the Earth’s magnetotail [4], while using a limited set of sensors.

The study of attitude estimation in formations is essential for their safety and the success of their mission. For instance, an accurate attitude estimate matched by an appropriate control system allows for close vehicle operations and for high-resolution interferometry. Nonetheless, such formations are still in the early stages of real-world application [5]. The ability to accurately and consistently estimate the attitude of a constrained formation increases the flexibility of the mission design, which potentially lowers costs and improves reliability.

Analyzing how noise affects estimated data is important for practical applications and enables the comparison between different algorithms, as in [6]. In practice, the deployment of any deterministic solution requires some knowledge or estimate of the respective uncertainty, because it indicates whether the solution is valid and hints at the corresponding accuracy. Then, such information can be applied in stochastic estimation schemes, such as the extended Kalman filter (EKF), which can further improve the results. For instance, the uncertainty of the attitude determination based on the Singular Value Decomposition (SVD) has recently been used to improve the accuracy and robustness of an implementation of the EKF [7]. Moreover, the covariance of an estimate can be used to establish the relative weight of the corresponding estimated value in sensor fusion, which combines estimates from different sources. The work presented herein is concerned with analyzing the error of the attitude estimation in a three-vehicle constrained formation whose solution was proposed in [8]. Such uncertainty analysis consists in associating each estimate with its covariance matrix.

The attitude of a system can be found resorting to memory, in which case it is called a filtering method, or, instead, by considering information available at a given instant, also known as a static method ([9], pp. 183–184), which is the case considered in this document. The first static attitude determination methods that considered vector observations were algebraic. One such method, which is still relevant today [10], is the Tri-Axial Attitude Determination (TRIAD) algorithm [11], whose uncertainty can be characterized considering an axially symmetric distribution for the error, while resorting to assumptions on the correlation of the measurements [12]. The TRIAD was later optimized in [13] by analyzing the variance of the error associated with each pair of observation vectors. However, its accuracy is limited because it only considers two independent measurements. The attitude determination was later framed as an optimization problem [14] by adding the positive determinant constraint to the orthogonal Procrustes problem [15]. This formulation potentially improves the accuracy of the solution because it allows the combination of data from as many sensors as desired. The original solution for the Wahba’s problem was given shortly after its publication [16]. The q-method improved the original solution by considering the quaternion representation of the attitude ([17], pp. 426–428). The Quaternion Estimator (QUEST) algorithm [12] provided a solution, which focused on computational efficiency, and its uncertainty can be characterized by using a plane tangent to the observation vector as an approximation for the error distribution, which is valid for small field-of-view (FOV) sensors [18]. An extension to such an uncertainty model for large FOV sensors can be found in [19]. As a result, it became and still is a popular attitude determination method, especially for real-time applications. Other quaternion-based methods include the Estimator of the Optimal Quaternion (ESOQ) [20], the Second Estimator of the Optimal Quaternion (ESOQ2) [21], the fast linear quaternion attitude estimator (FLAE) [22], and others such as [23], where the dot product equality constraint results in simplified covariance expressions for the quaternion solution. The matrix-based solution which applies the SVD is robust but computationally expensive [24], and its covariance makes some assumptions on the weights of the Wabha loss function. A faster matrix-based solution is given by the Fast Optimal Attitude Matrix (FOAM) [25]. The large number of proposed solutions for the Wabha problem is evidence in favor of the importance of this framework. Nonetheless, it does not consider all possible scenarios. The generalized Wahba problem tries to fill this gap by allowing attitude measurements [26], which is useful when many sensors are available.

Attitude estimation in constrained formations has been studied in [27], which considers a three-vehicle formation with three different sets of measurements, and [28], which considers a two-vehicle formation with a common landmark measurement. In such problems, the attitude is determined by sharing sensor data between the elements of the formation. Moreover, both works provide the covariance matrix for the respective solution, by considering the uncertainty model for wide FOV sensors [19] and assuming that the attitude errors are small. Additionally, [27] resorts to the Cramér–Rao inequality to find an upper bound for the covariance, whereas [28] uses the nonlinear least squares solution. Thus, it is important to add such study to the three-vehicle constrained formation from [8].

Therefore, this document is concerned with the uncertainty analysis of the attitude solution for the three-vehicle constrained formation proposed in [8], which has not been characterized yet. Consequently, the main contribution of this paper is the uncertainty characterization, which provides a theoretical value for the covariance of each relative and inertial attitude matrix of the formation in almost all configurations. Such values are obtained by considering first-order perturbations in the measurements used in the solution. This contribution is significant for the potential application of such an attitude determination method, because the precision data are essential for most conceivable applications and systems. Additionally, the uncertainty analysis is validated numerically by implementing an extensive set of Monte Carlo simulations and evaluating the respective results, both for regular configurations and near the special cases where such covariance is not valid, as predicted in [29].

In this document, Section 2 describes the notation used throughout the paper and briefly summarizes some important results applied in the uncertainty analysis. Then, Section 3 provides a complete description of the formation and attitude problem, followed by a summary of the solution proposed in [8] and the application of the weighted orthogonal Procrustes optimization—see [30,31]—to improve such a solution. The main results are given in Section 4, where the uncertainties of the estimates are analyzed by applying a first-order perturbation to the attitude solutions and adapting the existing work [32] for the covariance of the weighted orthogonal Procrustes optimization. The covariance expressions are derived in the same section for each of the attitude matrices. The section ends with some remarks regarding the uncertainty in the special configurations defined in [29]. Next, the uncertainty analysis is validated by comparing the results of the numerical implementation of the solution with the theoretical results for the covariance obtained in Section 4, which is performed resorting to a set of Monte Carlo simulations. Lastly, Section 6 gives some closing remarks about the results obtained in this work.

## 2. Preliminaries

### 2.1. Notation

Throughout the document, scalars are represented in regular typeface, whereas vectors and matrices are represented in bold, with the latter in capital case. The subscript $\left[i\right]$ denotes the *i*-th element of a vector or the *i*-th column of a matrix, accordingly. Moreover, in matrices, the subscript $\left[i,j\right]$ indicates the respective element at row *i* and column *j*. Reference frames are represented in calligraphic typeface and between brackets, such as I. Body-fixed frames are numbered and represented by the letter B, with the respective number as a subscript, whereas sensor-fixed frames are represented by the letter $S_{}$, with a subscript identifying the respective sensor. The symbol 0 represents the null vector or matrix, the symbol 1 denotes the vector with all entries equal to one, and I represents the identity matrix with the appropriate dimensions. Moreover, the expected value of a random variable is denoted by the operator $\langle .\rangle$.

The real coordinate space of dimension *N* is denoted by $R^{N}$. The set of unit vectors in $R^{3}$ is denoted by

$$S\left(2\right):=\left\{x\in R^{3}:∥x∥=1\right\}\;.$$

The special orthogonal group of dimension 3, which describes proper rotations, is denoted by

$$\mathrm{SO}\left(3\right):=\left\{X\in R^{3\times 3}:{\mathrm{XX}}^{T}=X^{T}X=I\;\wedge \;\det\left(X\right)=1\right\}\;.$$

Resorting to the passive transformation perspective, the rotation, in SO(3), that transforms the frame where a given vector, in $R^{3}$, is expressed from $B_{i}$ to $B_{j}$, i,j∈N, is denoted by $R_{i}^{j}$. If a frame is not body-fixed, its respective letter is used instead. For example, $R_{j}^{I}$ transforms the frame of a vector expressed in $B_{j}$ to the inertial frame. Moreover, multiple candidates for the same rotation are identified by a subscript capital case letter, such as ${\left(R_{i}^{j}\right)}_{A}$.

The trace of a matrix X is defined as $\mathrm{trace}\left(X\right)=\sum_{i=1}^{3}x_{ii}$, where $x_{ii}$ is the *i*-th element of the diagonal of X, and $\mathrm{diag}\left(X\right)$ denotes the diagonal matrix with identical diagonal entries to X. Moreover, the Frobenius norm of X is defined as $∥X∥=\sqrt{\mathrm{trace}\left(X^{T}X\right)}$ and the same notation is used for the Euclidean norm of a vector x, which is, respectively, defined as $∥x∥=\sqrt{x^{T}x}$.

The skew-symmetric matrix parameterized by $x\in R^{3}$, which encodes the cross product between x and another vector, is denoted by $S\left(x\right)$, such that $S^{{}^{-1}}\;\;\left(S\left(x\right)\right)=x$. The rotation matrix of an angle θ∈R about the axis described by the unit vector x∈S(2) is denoted by R(θ,x), which is written, recalling that the passive perspective is considered, as follows ([9], p. 42).

(1) $$R\left(\theta ,x\right):=\cos\left(\theta \right)I+\left(1-\cos\left(\theta \right)\right)x\;x^{T}-\sin\left(\theta \right)S\left(x\right)\;.$$

Several trigonometric functions are used throughout this document. Notably, the inverse cosine function is denoted by $\arccos\left(a\right)$, with $a\in R^{}$, and the four-quadrant inverse tangent function is denoted by $\mathrm{atan}2\left(b,a\right)$, with a,b∈R.

### 2.2. Useful Results

The following results are used in the uncertainty analysis. Let $x\in R^{3}$. Then,

(2) SxSx=xxT−xTxI.

and

(3) $$\mathrm{trace}\left(S\left(x\right)S\left(x\right)\right)=-2x^{T}x\;.$$

## 3. Deterministic Attitude Problem

### 3.1. Problem Definition

Consider a formation with three vehicles, where $B_{1}$, $B_{2}$, and $B_{3}$ are the body-fixed frames of the respective vehicles and I represents the inertial frame. In the proposed framework, there are two kinds of measurements: one is a line-of-sight (LOS) vector that points to the position of another vehicle, and the other is an inertial reference vector—for example, a known physical field direction. All measurements are unit vectors obtained in the respective body-fixed frame. Moreover, the inertial references are known in the inertial frame.

**Figure 1:**
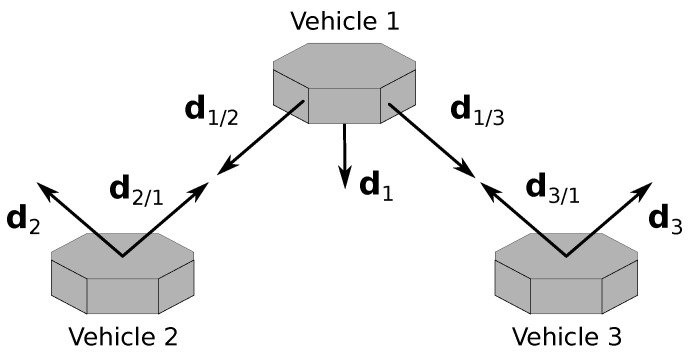
Three-vehicle heterogeneous formation.

In the formation, the main constraint is that two of the vehicles, called the deputies, cannot measure LOS vectors between them, meaning, for example, that these two vehicles are too far from each other. Furthermore, each vehicle can measure one inertial vector independently. The vehicle that measures LOS to the other two is identified as vehicle 1 and is denominated as the chief, whereas the deputies are identified as vehicles 2 and 3. The subgroup with the chief and a deputy is called a branch of the formation; hence, there are two branches. Branch 1–2 includes the chief and vehicle 2, whereas branch 1–3 includes the chief and vehicle 3. The geometry of the framework is represented in Figure 1.

Throughout this document, $d_{i/j}$, i,j=1,2,3, i≠j, represents the LOS vector from the *i*-th to the *j*-th vehicles, expressed in $B_{i}$, and $d_{i}$, i=1,2,3, represents the inertial vector measured by the *i*-th vehicle, expressed in $B_{i}$. A left superscript specifying the frame is used whenever a vector is expressed in a different frame. For example, $\;{\;}^{I}\;d_{j}$, j=1,2,3, is the inertial vector of the *j*-th vehicle, expressed in $I_{}$.

The problem that is here considered is that of determining all the rotation matrices, both relative ($R_{2}^{1}$, $R_{3}^{1}$, $R_{3}^{2}$) and inertial ($R_{1}^{I}$, $R_{2}^{I}$, $R_{3}^{I}$), using the measurement vectors that were described, as well as the references $\;\;{\;}^{I}\;d_{1}$, $\;{\;}^{I}\;d_{2}$, and $\;{\;}^{I}\;d_{3}$.

### 3.2. Solution

The solution proposed in [8] computes the inertial attitude of the chief using two different sets of data, one from each branch. The solution is found by choosing the attitudes which are consistent with both sets of information. First, the candidates for $R_{2}^{1}$ and $R_{3}^{1}$ are determined. Afterwards, these are used to obtain the candidates for $R_{1}^{I}$. Since computing $R_{1}^{I}$ using either $R_{2}^{1}$ or $R_{3}^{1}$ is equivalent, then it is possible to disambiguate the problem. Therefore, a comparison between the candidates for $R_{1}^{I}$ is carried out. Additionally, in the presence of sensor noise, the solutions for $R_{1}^{I}$ of both branches are combined using the orthogonal Procrustes problem to reduce the noise of the respective final value. Finally, the remaining matrices are found from the solutions for $R_{2}^{1}$, $R_{3}^{1}$, and $R_{1}^{I}$. A more detailed algorithm flowchart can be found in [8].

In the following summary, it is assumed that the configurations have a unique solution, as described in [29].

#### 3.2.1. Relative Attitude

This section describes the expressions which result in the candidates for $R_{2}^{1}$. The expressions for branch 1–3 are omitted because these are completely analogous. The ensuing derivation also relates to the work in [28], where it is shown that using a planar constraint leads to an ambiguity. In this case, however, the problem is not constrained to a triangle, and, therefore, such ambiguity cannot be resolved without extra information, which will be provided when combining the information of both branches. Hence, recall the parameterization (1) and consider the decomposition of the relative attitude given by

(4) $$R_{2}^{1}:=R\left(\theta_{2},n_{2}\right)R\left(\theta_{1},n_{1}\right)\;,$$

with $\theta_{1},\theta_{2}\in R$ and $n_{1},n_{2}\in S\left(2\right)$, such that $R_{2}^{1}$ verifies the constraints expressed as $-d_{1/2}=R_{2}^{1}d_{2/1}$ and $d_{1}^{T}R_{2}^{1}d_{2}=\;{\;}^{I}\;d_{1}^{T}\;\;\;{\;}^{I}\;d_{2}$. The resulting parameters are given by

(5a) $$\theta_{1}:=\pi \;,$$

(5b) $$\left\{\begin{array}{cc} n_{1}:=\frac{d_{2/1}-d_{1/2}}{∥d_{2/1}-d_{1/2}∥}\; & ,\;\mathrm{for}\;d_{2/1}\neq d_{1/2}\\ n_{1}:=\frac{S\left(d_{1/2}\right)d_{1}}{∥S\left(d_{1/2}\right)d_{1}∥}\; & ,\;\mathrm{for}\;d_{2/1}=d_{1/2} \end{array}\right.\;,$$

(6a) $$\theta_{2}:=\mathrm{atan}2\left(c_{s_{12}},c_{c_{12}}\right)+\sigma_{12}\arccos\left(\frac{c_{p_{12}}}{\sqrt{c_{s_{12}}^{2}+c_{c_{12}}^{2}}}\right)\;,$$

and

(6b) $$n_{2}:=-d_{1/2}\;,$$

with $\sigma_{12}\in \left\{-1,+1\right\}$ and

(7) $$\left\{\begin{array}{cc} c_{p_{12}} & :=d_{1}^{T}\left(d_{1/2}\right){\left(d_{1/2}\right)}^{T}d_{2}^{⋆}-\;{\;}^{I}\;d_{1}^{T}\;\;{\;}^{I}\;d_{2}\\ c_{c_{12}} & :=d_{1}^{T}S{\left(d_{1/2}\right)}^{2}d_{2}^{⋆}\\ c_{s_{12}} & :=d_{1}^{T}S\left(-d_{1/2}\right)d_{2}^{⋆} \end{array}\right.\;,$$

where

(8) $$d_{2}^{⋆}:=R\left(\theta_{1},n_{1}\right)d_{2}\;.$$

There are, in general, two candidates for $R_{2}^{1}$. Such ambiguity of the solution is condensed in $\sigma_{12}$, which carries the choice made by the algorithm after the disambiguation and is useful to the covariance computation. The same reasoning applies to $R_{3}^{1}$.

#### 3.2.2. Inertial Attitude Candidate

Considering the data in branch 1–2, the constraints on $R_{1}^{I}$ are given by

$$\;{\;}^{I}\;d_{1}=R_{1}^{I}d_{1}$$

and

$$\;{\;}^{I}\;d_{2}=R_{1}^{I}R_{2}^{1}d_{2}\;.$$

Since these constraints imply that there are two pairs of vectors represented in two different coordinate frames, assuming that these vectors are non-collinear, then the candidates for $R_{1}^{I}$ are computed using the TRIAD algorithm [11]. Therefore, the direct application of the TRIAD results in ${\left(R_{1}^{I}\right)}_{A}$ and ${\left(R_{1}^{I}\right)}_{B}$, if $\;{\;}^{I}\;d_{1}\neq \pm \;{\;}^{I}\;d_{2}$. Indeed, from the first branch

(9) $$\begin{array}{c} R_{1}^{I}=\;{\;}^{I}\;d_{1}d_{1}^{T}+\frac{S\left(\;{\;}^{I}\;d_{1}\right)\;{\;}^{I}\;d_{2}}{∥S\left(\;{\;}^{I}\;d_{1}\right)\;{\;}^{I}\;d_{2}∥}{\left(\frac{S\left(d_{1}\right)R_{2}^{1}d_{2}}{∥S\left(d_{1}\right)R_{2}^{1}d_{2}∥}\right)}^{T}\;\;\;\;\;\;\;\;\;\;\;\\ \;\;\;\;\;\;\;\;\;\;\;\;+\left(S\left(\;{\;}^{I}\;d_{1}\right)\frac{S\left(\;{\;}^{I}\;d_{1}\right)\;{\;}^{I}\;d_{2}}{∥S\left(\;{\;}^{I}\;d_{1}\right)\;{\;}^{I}\;d_{2}∥}\right){\left(S\left(d_{1}\right)\frac{S\left(d_{1}\right)R_{2}^{1}d_{2}}{∥S\left(d_{1}\right)R_{2}^{1}d_{2}∥}\right)}^{T}\;, \end{array}$$

where ${\left(R_{1}^{I}\right)}_{A}$ and ${\left(R_{1}^{I}\right)}_{B}$ are obtained by replacing $R_{2}^{1}$ with ${\left(R_{2}^{1}\right)}_{A}$ and ${\left(R_{2}^{1}\right)}_{B}$, respectively.

Similarly, ${\left(R_{1}^{I}\right)}_{C}$ and ${\left(R_{1}^{I}\right)}_{D}$ are obtained by applying the term analogous to (9) for branch 1–3 and replacing $R_{3}^{1}$ with ${\left(R_{3}^{1}\right)}_{C}$ and ${\left(R_{3}^{1}\right)}_{D}$, respectively.

#### 3.2.3. Comparison

Recalling that the configuration is assumed non-degenerate; then, from the four candidates for $R_{1}^{I}$, there is a pair of identical matrices emerging from the two different branches. This means that at least one of the following equations must be verified:

$${\left(R_{1}^{I}\right)}_{A}={\left(R_{1}^{I}\right)}_{C}\;,\;{\left(R_{1}^{I}\right)}_{A}={\left(R_{1}^{I}\right)}_{D}\;,\;{\left(R_{1}^{I}\right)}_{B}={\left(R_{1}^{I}\right)}_{C}\;,\;\mathrm{or}\;{\left(R_{1}^{I}\right)}_{B}={\left(R_{1}^{I}\right)}_{D}\;.$$

The comparison between candidates is made resorting to the rotation defined by

(10) $${\left(R_{1}^{I}\right)}_{XY}:={\left(R_{1}^{I}\right)}_{X}{\left(R_{1}^{I}\right)}_{Y}^{T}\;,$$

where ${\left(R_{1}^{I}\right)}_{X}$ and ${\left(R_{1}^{I}\right)}_{Y}$ represent different candidates. This rotation is an identity matrix when $\left(R_{1}^{I}\right){\;}_{X}$ and $\left(R_{1}^{I}\right){\;}_{Y}$ are identical. In such a case, the principal angle of ${\left(R_{1}^{I}\right)}_{XY}$ is zero, and, therefore, its absolute value is used as the comparison parameter that gives the proximity between each pair of candidates for $R_{1}^{I}$.

The trace is used to find the principal angle of (10) because the trace of a square matrix is the sum of its eigenvalues, which, in this case, is given as $\mathrm{trace}\left({\left(R_{1}^{I}\right)}_{XY}\right)=1+2\cos\left(\theta_{XY}\right)$, where $\theta_{XY}$ is the principal angle of ${\left(R_{1}^{I}\right)}_{XY}$. Then, using $\mu_{}$ to denote the absolute value of the principal angle, i.e., $\mu_{}:=\left|\theta_{}\right|$, and rearranging the equation, it follows that the comparison parameter is expressed as

(11) $$\mu_{XY}=\left|\arccos\left(\frac{\mathrm{trace}\left({\left(R_{1}^{I}\right)}_{XY}\right)-1}{2}\right)\right|\;.$$

#### 3.2.4. Complete Solution

The remaining attitude matrices, i.e., $R_{3}^{2}$, $R_{2}^{I}$, and $R_{3}^{I}$, are obtained from a product between the attitudes already determined, given as

(12a) $$R_{2}^{I}=R_{1}^{I}R_{2}^{1}\;,$$

(12b) $$R_{3}^{I}=R_{1}^{I}R_{3}^{1}\;,$$

and

(12c) $$R_{3}^{2}=R_{2}^{1}\;{\;}^{T}R_{3}^{1}\;.$$

#### 3.2.5. Sensor Errors

In the presence of sensor errors, the inertial candidates do not match exactly, in general. Hence, the values of $\mu_{}$ are, in general, different from zero. In this case, the solution is given by the smallest $\mu_{}$. Nonetheless, the smallest value of $\mu_{}$ includes two different candidates for $R_{1}^{I}$ due to noise errors. The final value for this inertial attitude is found by solving the weighted orthogonal Procrustes problem as described in [30,31]. Thus far, the covariance is unknown, in which case all weights are set to be identical and the result is an average matrix. However, the covariance matrices, defined in the uncertainty analysis given in the next section, allow an optimization by considering the uncertainty of each candidate in the weight choice. The weighted orthogonal Procrustes problem finds the value of $R_{1}^{I}$ which minimizes the cost function given as

(13) $$c\left(R_{1}^{I}\right):={∥\left(A-R_{1}^{I}B\right)G^{-1/2}∥}^{2}\;,$$

where A is the matrix which combines both inertial candidates, as defined by

(14) $$A:=\left[\begin{array}{cc} {\left(R_{1}^{I}\right)}_{X} & {\left(R_{1}^{I}\right)}_{Y} \end{array}\right]\;,$$

B is the matrix with the respective references, as defined by

(15) $$B:=\left[\begin{array}{cc} I & I \end{array}\right]\;,$$

and G is a diagonal matrix whose entries are the problem weights and is given as

$$G=\mathrm{diag}\left(\sigma_{1}^{2},\;\sigma_{2}^{2},\;\sigma_{3}^{2},\;\sigma_{4}^{2},\;\sigma_{5}^{2},\;\sigma_{6}^{2}\right)\;.$$

Such weights are defined as the maximum eigenvalues of $\Sigma_{a_{\left[i\right]}}$, which denotes the covariance matrix of the *i*-th column of A. Hence,

(16) $$\sigma_{i}^{2}:=\lambda_{\max}\left(\Sigma_{a_{\left[i\right]}}\right)\;\mathrm{for}\;i=1,2,3,4,5,6\;.$$

The solution of the weighted orthogonal Procrustes problem is determined from the weighted covariance between observations and references, which is computed resorting to the symmetric weight matrix given as $W=G^{-1}-\frac{1}{n_{w}}G^{-1}11^{T}G^{-1}$ with $n_{w}=1^{T}G{}^{-1}1$. Therefore, considering the SVD expressed as $AWB^{T}=UDV^{T}$, it follows that the solution for $R_{1}^{I}$ is given by

$$R_{1}^{I}=U\left[\begin{array}{ccc} 1 & 0 & 0\\ 0 & 1 & 0\\ 0 & 0 & \det\left(UV^{T}\right) \end{array}\right]V^{T}\;.$$

## 4. Uncertainty Analysis

The uncertainty analysis is divided into three parts: the analysis of the candidates of a single branch, the analysis of the combined solution for $R_{1}^{I}$, and the analysis of the solutions for $R_{2}^{I}$, $R_{3}^{I}$, and $R_{3}^{2}$. The first implements a first-order perturbation and obtains the respective covariance matrices considering the linearization of the solutions. It is assumed that the perturbation is small enough for the linearization to be valid. The second applies the weighted orthogonal Procrustes uncertainty analysis, which has been reported in the literature and follows the same principles of linear perturbation; see [32]. Lastly, the third part applies the covariance definition and resorts to the results of the previous two parts.

### 4.1. Analysis of a Branch

In this section, consider the branch 1–2 and the respective candidates for $R_{2}^{1}$ and $R_{1}^{I}$. Moreover, denote the inertial candidate of branch 1–2 which minimizes $\mu_{}$, from (11), as ${\left(R_{1}^{I}\right)}_{X}$. The ensuing analysis is analogous to that of branch 1–3, and, therefore, the respective expressions for the covariance matrices of such a branch are easily obtained from the results described hereafter.

The noise analysis of the branch attitude candidates considers a first-order perturbation in the measurements made by the sensors. Such perturbation propagates through all the operations required to obtain each of the attitude matrices and is reflected in their errors. Then, the perturbation at the level of the attitude is used to compute the respective covariance matrix, which summarizes the expected first-order errors for a particular set of measurements disturbed by noise following a known distribution.

At the level of the measurements, consider the perturbations described by the following error models

(17a) $$d_{1}=d_{1}{\;}^{\left(0\right)}+\epsilon \;d_{1}{\;}^{\left(1\right)}+O\left(\epsilon^{2}\right)\;,$$

(17b) $$d_{2}=d_{2}{\;}^{\left(0\right)}+\epsilon \;d_{2}{\;}^{\left(1\right)}+O\left(\epsilon^{2}\right)\;,$$

(17c) $$d_{12}=d_{12}{\;}^{\left(0\right)}+\epsilon \;d_{12}{\;}^{\left(1\right)}+O\left(\epsilon^{2}\right)\;,$$

and

(17d) $$d_{21}=d_{21}{\;}^{\left(0\right)}+\epsilon \;d_{21}{\;}^{\left(1\right)}+O\left(\epsilon^{2}\right)\;,$$

where ϵ denotes a smallness parameter, $\left(.\right){\;}^{\left(0\right)}$ denotes the zeroth-order terms, $\left(.\right){\;}^{\left(1\right)}$ denotes the first-order terms, and $O\left(\epsilon^{2}\right)$ denotes the higher-order terms. Moreover, the first-order errors are assumed to follow zero mean known distributions and the respective covariance matrices are defined as

$$\Sigma_{1}:=\langle d_{1}{\;}^{\left(1\right)}{\left(d_{1}{\;}^{\left(1\right)}\right)}^{T}\rangle \;,$$

$$\Sigma_{2}:=\langle d_{2}{\;}^{\left(1\right)}{\left(d_{2}{\;}^{\left(1\right)}\right)}^{T}\rangle \;,$$

$$\Sigma_{12}:=\langle d_{12}{\;}^{\left(1\right)}{\left(d_{12}{\;}^{\left(1\right)}\right)}^{T}\rangle$$

and

$$\Sigma_{21}:=\langle d_{21}{\;}^{\left(1\right)}{\left(d_{21}{\;}^{\left(1\right)}\right)}^{T}\rangle \;.$$

Next, consider the analogous perturbation model applied to the attitude matrices of the branch, as given by

(18a) $$R_{2}^{1}=R_{2}^{1}{\;}^{\left(0\right)}+\epsilon R_{2}^{1}{\;}^{\left(1\right)}+O\left(\epsilon^{2}\right)\;,$$

and

(18b) $${\left(R_{1}^{I}\right)}_{X}={\left(R_{1}^{I}\right)}_{X}^{\left(0\right)}+\epsilon {\left(R_{1}^{I}\right)}_{X}^{\left(1\right)}+O\left(\epsilon^{2}\right)\;,$$

with the respective covariance matrices defined as

(19a) $$\Sigma_{R12}:=\langle R_{2}^{1}{\;}^{\left(1\right)}{\left(R_{2}^{1}{\;}^{\left(1\right)}\right)}^{T}\rangle \;,$$

and

(19b) $$\Sigma_{Rx}:=\langle {\left(R_{1}^{I}\right)}_{X}^{\left(1\right)}{\left({\left(R_{1}^{I}\right)}_{X}^{\left(1\right)}\right)}^{T}\rangle \;.$$

**Assumption** **1.** *The first-order perturbations of the measurements are small enough, such that the perturbations induced in the attitude are approximately linear.*

Assumption 1 guarantees that the expected values of $R_{2}^{1}{\;}^{\left(0\right)}$ and ${\left(R_{1}^{I}\right)}_{X}^{\left(0\right)}$, as defined in (18), are equal to their respective true values. As a result, the solutions for $R_{2}^{1}$ and ${\left(R_{1}^{I}\right)}_{X}$ can be linearized using a first-order Taylor approximation, provided that the respective Jacobian matrices are well defined. Moreover, the covariance matrices in (19) are computed from the propagation of the measurement perturbations in the linearized solution. The cases where the Jacobian is not well defined are associated with degenerate or coplanar configurations, as will be concluded in the sequel. Finally, the numerical simulations in Section 5 validate Assumption 1 for a typical sensor noise value.

#### 4.1.1. Linearized Solution

Consider the vector of combined measurements required in branch 1–2 given by

$$z_{2}:={\left[\begin{array}{cccc} d_{1}^{T}\; & d_{1/2}^{T}\; & d_{2/1}^{T}\; & d_{2}^{T} \end{array}\right]}^{T}\;,$$

and recall the Taylor expansion of a function. Since the attitude matrices can be viewed as a set of 3 vectors each with 3 functions, then the linearization of the *i*-th column of $R_{2}^{1}$ is given as

(20) $$R_{2\left[i\right]}^{1}\left(z_{2}\right)=R_{2\left[i\right]}^{1\;\left(0\right)}+DR_{2\left[i\right]}^{1}\left(z_{2}-z_{2}{\;}^{\left(0\right)}\right)$$

with $R_{2\left[i\right]}^{1}\left(z_{2}\right)$ denoting the *i*-th column of the attitude matrix and the respective Jacobian matrix defined as

$$DR_{2\left[i\right]}^{1}:=\left[\begin{array}{ccccccccc} \frac{\partial R_{2\left[i\right]}^{1}}{\partial d_{1\left[1\right]}}\; & ...\; & \frac{\partial R_{2\left[i\right]}^{1}}{\partial d_{1/2\left[1\right]}}\; & ...\; & \frac{\partial R_{2\left[i\right]}^{1}}{\partial d_{2/1\left[1\right]}}\; & ...\; & \frac{\partial R_{2\left[i\right]}^{1}}{\partial d_{2\left[1\right]}}\; & \frac{\partial R_{2\left[i\right]}^{1}}{\partial d_{2\left[2\right]}}\; & \frac{\partial R_{2\left[i\right]}^{1}}{\partial d_{2\left[3\right]}} \end{array}\right]\;,$$

where $d_{1\left[1\right]}$ denotes the first element of $d_{1}$ and analogously for the remaining measurements.

Recall (4) and the respective expressions for each of its parameters given in (5)–(7). Then, the dependence of the relative attitude on the measurements can be tracked by drawing a tree of the variables involved in the computation of each parameter as follows:


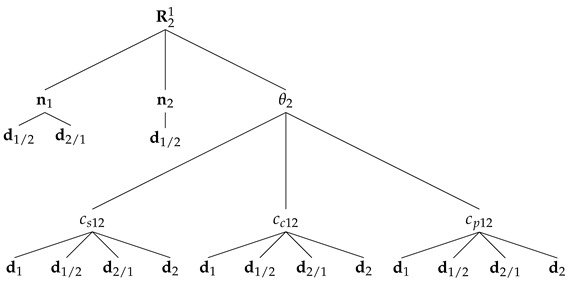


Then, recalling the chain rule of the partial derivatives, it follows that

(21a) $$\frac{\partial R_{2}^{1}}{\partial d_{1\left[j\right]}}=\frac{\partial R_{2}^{1}}{\partial \theta_{2}}\left(\frac{\partial \theta_{2}}{\partial c_{s12}}\frac{\partial c_{s12}}{\partial d_{1\left[j\right]}}+\frac{\partial \theta_{2}}{\partial c_{c12}}\frac{\partial c_{c12}}{\partial d_{1\left[j\right]}}+\frac{\partial \theta_{2}}{\partial c_{p12}}\frac{\partial c_{p12}}{\partial d_{1\left[j\right]}}\right)\;,$$

(21b) $$\begin{array}{c} \frac{\partial R_{2}^{1}}{\partial d_{12\left[j\right]}}=\sum_{k=1}^{3}\frac{\partial R_{2}^{1}}{\partial n_{1\left[k\right]}}\frac{\partial n_{1\left[k\right]}}{\partial d_{12\left[j\right]}}+\frac{\partial R_{2}^{1}}{\partial n_{2\left[k\right]}}\frac{\partial n_{2\left[k\right]}}{\partial d_{12\left[j\right]}}\;\;\;\;\;\;\;\;\;\;\;\\ \;\;\;\;\;\;\;\;\;\;\;\;+\frac{\partial R_{2}^{1}}{\partial \theta_{2}}\left(\frac{\partial \theta_{2}}{\partial c_{s12}}\frac{\partial c_{s12}}{\partial d_{12\left[j\right]}}+\frac{\partial \theta_{2}}{\partial c_{c12}}\frac{\partial c_{c12}}{\partial d_{12\left[j\right]}}+\frac{\partial \theta_{2}}{\partial c_{p12}}\frac{\partial c_{p12}}{\partial d_{12\left[j\right]}}\right)\;, \end{array}$$

(21c) $$\frac{\partial R_{2}^{1}}{\partial d_{21\left[j\right]}}=\sum_{k=1}^{3}\frac{\partial R_{2}^{1}}{\partial n_{1\left[k\right]}}\frac{\partial n_{1\left[k\right]}}{\partial d_{21\left[j\right]}}+\frac{\partial R_{2}^{1}}{\partial \theta_{2}}\left(\frac{\partial \theta_{2}}{\partial c_{s12}}\frac{\partial c_{s12}}{\partial d_{21\left[j\right]}}+\frac{\partial \theta_{2}}{\partial c_{c12}}\frac{\partial c_{c12}}{\partial d_{21\left[j\right]}}+\frac{\partial \theta_{2}}{\partial c_{p12}}\frac{\partial c_{p12}}{\partial d_{21\left[j\right]}}\right)\;,$$

and

(21d) $$\frac{\partial R_{2}^{1}}{\partial d_{2\left[j\right]}}=\frac{\partial R_{2}^{1}}{\partial \theta_{2}}\left(\frac{\partial \theta_{2}}{\partial c_{s12}}\frac{\partial c_{s12}}{\partial d_{2\left[j\right]}}+\frac{\partial \theta_{2}}{\partial c_{c12}}\frac{\partial c_{c12}}{\partial d_{2\left[j\right]}}+\frac{\partial \theta_{2}}{\partial c_{p12}}\frac{\partial c_{p12}}{\partial d_{2\left[j\right]}}\right)\;.$$

All the partial derivatives expressed in (21) are given in Appendix A and are obtained directly from the relative attitude solution given in Section 3.

Similarly, using the $R_{x\left[i\right]}^{}$ to denote the *i*-th column of ${\left(R_{1}^{I}\right)}_{X}$, the column-wise linearization of ${\left(R_{1}^{I}\right)}_{X}$ is given by

(22) $$R_{x\left[i\right]}^{}\left(z_{2}\right)=R_{x\left[1\right]}^{\;\left(0\right)}\;+DR_{x\left[i\right]}^{}\left(z_{2}-z_{2}{\;}^{\left(0\right)}\right)$$

with $R_{x\left[i\right]}^{}\left(z_{2}\right)$ denoting the *i*-th column of the attitude matrix and the respective Jacobian matrix defined as

(23) $$DR_{x\left[i\right]}^{}:=\left[\begin{array}{ccccccc} \frac{\partial R_{x\left[i\right]}^{}}{\partial d_{1\left[1\right]}}\; & ...\; & \frac{\partial R_{x\left[i\right]}^{}}{\partial d_{1/2\left[1\right]}}\; & ...\; & \frac{\partial R_{x\left[i\right]}^{}}{\partial d_{2/1\left[1\right]}}\; & ...\; & \frac{\partial R_{x\left[i\right]}^{}}{\partial d_{2\left[3\right]}} \end{array}\right]\;.$$

Recalling (9) and that $R_{2}^{1}$ is computed from (4), it follows that the dependence of the inertial candidate of branch 1–2 on the measurements can be tracked by drawing a tree of variables, analogous to the one concerning $R_{2}^{1}$, as follows:


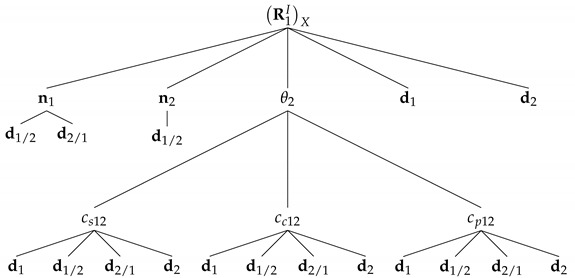


Then, recalling the chain rule of the partial derivatives, it follows that

(24a) $$\frac{\partial {\left(R_{1}^{I}\right)}_{X}}{\partial d_{1\left[j\right]}}=\frac{\partial {\left(R_{1}^{I}\right)}_{X}\left(z_{2}\right)}{\partial d_{1\left[j\right]}}+\frac{\partial {\left(R_{1}^{I}\right)}_{X}}{\partial \theta_{2}}\left(\frac{\partial \theta_{2}}{\partial c_{s12}}\frac{\partial c_{s12}}{\partial d_{1\left[j\right]}}+\frac{\partial \theta_{2}}{\partial c_{c12}}\frac{\partial c_{c12}}{\partial d_{1\left[j\right]}}+\frac{\partial \theta_{2}}{\partial c_{p12}}\frac{\partial c_{p12}}{\partial d_{1\left[j\right]}}\right)\;,$$

(24b) $$\begin{array}{c} \frac{\partial {\left(R_{1}^{I}\right)}_{X}}{\partial d_{12\left[j\right]}}=\sum_{k=1}^{3}\frac{\partial {\left(R_{1}^{I}\right)}_{X}}{\partial n_{1\left[k\right]}}\frac{\partial n_{1\left[k\right]}}{\partial d_{12\left[j\right]}}+\frac{\partial {\left(R_{1}^{I}\right)}_{X}}{\partial n_{2\left[k\right]}}\frac{\partial n_{2\left[k\right]}}{\partial d_{12\left[j\right]}}\;\;\;\;\;\;\;\;\\ \;\;\;\;\;\;\;\;+\frac{\partial {\left(R_{1}^{I}\right)}_{X}}{\partial \theta_{2}}\left(\frac{\partial \theta_{2}}{\partial c_{s12}}\frac{\partial c_{s12}}{\partial d_{12\left[j\right]}}+\frac{\partial \theta_{2}}{\partial c_{c12}}\frac{\partial c_{c12}}{\partial d_{12\left[j\right]}}+\frac{\partial \theta_{2}}{\partial c_{p12}}\frac{\partial c_{p12}}{\partial d_{12\left[j\right]}}\right)\;, \end{array}$$

(24c) $$\begin{array}{c} \frac{\partial {\left(R_{1}^{I}\right)}_{X}}{\partial d_{21\left[j\right]}}=\sum_{k=1}^{3}\frac{\partial {\left(R_{1}^{I}\right)}_{X}}{\partial n_{1\left[k\right]}}\frac{\partial n_{1\left[k\right]}}{\partial d_{21\left[j\right]}}\;\;\;\;\;\;\;\;\;\\ \;\;\;\;\;\;\;\;\;+\frac{\partial {\left(R_{1}^{I}\right)}_{X}}{\partial \theta_{2}}\left(\frac{\partial \theta_{2}}{\partial c_{s12}}\frac{\partial c_{s12}}{\partial d_{21\left[j\right]}}+\frac{\partial \theta_{2}}{\partial c_{c12}}\frac{\partial c_{c12}}{\partial d_{21\left[j\right]}}+\frac{\partial \theta_{2}}{\partial c_{p12}}\frac{\partial c_{p12}}{\partial d_{21\left[j\right]}}\right)\;, \end{array}$$

and

(24d) $$\frac{\partial {\left(R_{1}^{I}\right)}_{X}}{\partial d_{2\left[j\right]}}=\frac{\partial {\left(R_{1}^{I}\right)}_{X}\left(z_{2}\right)}{\partial d_{2\left[j\right]}}+\frac{\partial {\left(R_{1}^{I}\right)}_{X}}{\partial \theta_{2}}\left(\frac{\partial \theta_{2}}{\partial c_{s12}}\frac{\partial c_{s12}}{\partial d_{2\left[j\right]}}+\frac{\partial \theta_{2}}{\partial c_{c12}}\frac{\partial c_{c12}}{\partial d_{2\left[j\right]}}+\frac{\partial \theta_{2}}{\partial c_{p12}}\frac{\partial c_{p12}}{\partial d_{2\left[j\right]}}\right)\;.$$

Again, all the partial derivatives expressed in (24) are given in Appendix A and are obtained directly from the relative attitude solution given in Section 3.

#### 4.1.2. Covariance Matrices

Recalling the definition of the covariance matrices in (19a) and (19b), it is concluded that the covariance of a rotation matrix is the sum of the covariance of the respective columns. Therefore, we substitute the first-order perturbations in (20) and (22) which, recalling the matrix multiplication properties and that the expected value is a linear operator, yield

(25a) ΣR12=∑i=13DR2i1z21z21TDR2i1T.

and

(25b) $$\Sigma_{Rx}=\sum_{i=1}^{3}DR_{x\left[i\right]}^{}\langle z_{2}{\;}^{\left(1\right)}{\left(z_{2}{\;}^{\left(1\right)}\right)}^{T}\rangle {\left(DR_{x\left[i\right]}^{I}\right)}^{T}\;,$$

where

(26) $$\langle z_{2}{\;}^{\left(1\right)}{\left(z_{2}{\;}^{\left(1\right)}\right)}^{T}\rangle =\left[\begin{array}{cccc} \Sigma_{1} & 0 & 0 & 0\\ 0 & \Sigma_{12} & 0 & 0\\ 0 & 0 & \Sigma_{21} & 0\\ 0 & 0 & 0 & \Sigma_{2} \end{array}\right]\;.$$

The analogous matrices for branch 1–3, i.e., $\Sigma_{R13}$ and $\Sigma_{Ry}$, are computed using completely analogous expressions.

#### 4.1.3. Covariance of Error Rotation Vector

The rotation vector of the attitude error denotes the axis and magnitude of such error. Since it is an intuitive representation for the error, the simulation results are expressed as rotation vectors, and, hence, it is convenient to denote the theoretical covariance of such a variable for comparison.

Consider $R_{2}^{1}$ and assume small errors. Then, considering the first-order errors, the attitude error matrix is approximately given by [9] (p. 59) $R_{2}^{1}\approx \left(I-S\left(\delta \theta_{12}\right)\right)R_{2}^{1}{\;}^{\left(0\right)}$, where $\delta \theta_{12}$ denotes the rotation vector of the error. Therefore, recalling the perturbation model in (18) and properties (2) and (3), the covariance with respect to $\delta \theta_{12}$ is given by

(27) $$\Sigma_{\delta \theta_{12}}=-\left[\Sigma_{R12}+\frac{1}{2}\mathrm{trace}\left(\Sigma_{R12}\right)I\right]\;.$$

Analogous transformations yield $\Sigma_{\delta \theta_{13}}$, $\Sigma_{\delta \theta_{x}}$, and $\Sigma_{\delta \theta_{y}}$. Furthermore, the inverse transformation is expressed as

(28) $$\Sigma_{R12}=-\Sigma_{\delta \theta_{12}}+\mathrm{trace}\left(\Sigma_{\delta \theta_{12}}\right)I\;.$$

### 4.2. Analysis of the Procrustes Optimization

Before considering the covariance associated with $R_{1}^{I}$, it is convenient to define the analogous term of $z_{2}$ which, for branch 1–3, is defined as

$$z_{3}:={\left[\begin{array}{cccc} d_{1}^{T}\; & d_{1/3}^{T}\; & d_{3/1}^{T}\; & d_{3}^{T} \end{array}\right]}^{T}\;.$$

As a consequence of the independence of measurements, the cross-covariance between $z_{2}$ and $z_{3}$ is given as

(29) $$\langle z_{2}{\;}^{\left(1\right)}{\left(z_{3}{\;}^{\left(1\right)}\right)}^{T}\rangle =\left[\begin{array}{cccc} \Sigma_{1} & 0 & 0 & 0\\ 0 & 0 & 0 & 0\\ 0 & 0 & 0 & 0\\ 0 & 0 & 0 & 0 \end{array}\right]\;.$$

Moreover, denote the *i*-th column of ${\left(R_{1}^{I}\right)}_{Y}$ as $R_{y\left[i\right]}^{}$ and recall that $R_{x\left[i\right]}^{}$ represents the *i*-th column of ${\left(R_{1}^{I}\right)}_{X}$. Then, the respective cross-covariance is defined as

(30) $$\langle R_{x\left[i\right]}^{\;\left(1\right)}{\left(R_{y\left[j\right]}^{\;\left(1\right)}\right)}^{T}\rangle =DR_{x\left[i\right]}^{}\langle z_{2}{\;}^{\left(1\right)}{\left(z_{3}{\;}^{\left(1\right)}\right)}^{T}\rangle {\left(DR_{y\left[j\right]}^{}\right)}^{T}\;,$$

with $DR_{x\left[i\right]}^{}$ defined in (23) and $DR_{y\left[i\right]}^{}$ being its analogous term for branch 1–3.

Next, recall that in the solution described in Section 3, the algorithm chooses the pair of branch candidates which are most consistent with each other, after having compared all candidates for $R_{1}^{I}$. Nonetheless, there are two distinct values for $R_{1}^{I}$ in the presence of noise, and therefore, the final result is given by the solution of the weighted orthogonal Procrustes problem—that is, the rotation that minimizes the cost expressed in (13). The covariance for such a problem is considered in [32] and is given with respect to the rotation vector of the error. Nonetheless, such covariance can be converted to the analogous term of (19a) and (19b) by applying (28). Hence, recall that

(31) $$R_{1}^{I}\approx \left(I-S\left(\delta \theta_{i1}\right)\right)R_{1}^{I}{\;}^{\left(0\right)}\;,$$

and that the covariance with respect to the rotation vector is defined as $\Sigma_{\delta \theta_{i1}}=\langle \delta \theta_{i1}\delta \theta_{i1}^{T}\rangle$. In [32], it is shown that $R_{1}^{I}{\;}^{\left(0\right)}$ corresponds to the true value of the attitude, which is valid here because $R_{2}^{1}{\;}^{\left(0\right)}$ and ${\left(R_{1}^{I}\right)}_{X}^{\left(0\right)}$ were shown to be the true values and the analogous conclusion is made for $R_{3}^{1}{\;}^{\left(0\right)}$ and ${\left(R_{1}^{I}\right)}_{Y}^{\left(0\right)}$. Then, from (15), B has zero uncertainty, which simplifies the covariance matrix described in [32] to

(32) $$\Sigma_{\delta \theta_{i1}}=H^{-1}\left[\sum_{i=1}^{6}\sum_{j=1}^{6}\sigma_{i}^{-2}\sigma_{j}^{-2}S\left(R_{1}^{I}{\;}^{\left(0\right)}b_{\left[i\right]}\right)\langle {\bar{a}}_{\left[i\right]}^{\left(1\right)}{\left({\bar{a}}_{\left[j\right]}^{\left(1\right)}\right)}^{T}\rangle S\left(R_{1}^{I}{\;}^{\left(0\right)}b_{\left[j\right]}\right)\right]H^{-1^{T}}$$

where $\sigma_{i}$ denotes the *i*-th weight as defined in (16), $b_{\left[i\right]}$ is the *i*-th column of B, as defined in (15), the auxiliary matrix H is given by

$$H:=-\sum_{i=1}^{6}\sigma_{i}^{-2}S\left(R_{1}^{I}{\;}^{\left(0\right)}b_{\left[i\right]}\right)S{\left(R_{1}^{I}{\;}^{\left(0\right)}b_{\left[i\right]}\right)}^{T}+\frac{1}{n_{w}}\sum_{i=1}^{6}\sum_{j=1}^{6}\sigma_{i}^{-2}\sigma_{j}^{-2}S\left(R_{1}^{I}{\;}^{\left(0\right)}b_{\left[i\right]}\right)S{\left(R_{1}^{I}{\;}^{\left(0\right)}b_{\left[j\right]}\right)}^{T}\;,$$

and the expected values in (32) are defined as

$$\begin{array}{c} \langle {\bar{a}}_{\left[i\right]}^{\left(1\right)}{\left({\bar{a}}_{\left[j\right]}^{\left(1\right)}\right)}^{T}\rangle =\langle a_{\left[i\right]}^{\left(1\right)}{\left(a_{\left[j\right]}^{\left(1\right)}\right)}^{T}\rangle -\frac{1}{n_{w}}\sum_{k=1}^{6}\sigma_{k}^{-2}\langle a_{\left[k\right]}^{\left(1\right)}{\left(a_{\left[j\right]}^{\left(1\right)}\right)}^{T}\rangle \;\;\;\;\;\;\;\;\\ \;\;\;\;\;\;\;\;-\frac{1}{n_{w}}\sum_{k=1}^{6}\sigma_{k}^{-2}\langle a_{\left[i\right]}^{\left(1\right)}{\left(a_{\left[k\right]}^{\left(1\right)}\right)}^{T}\rangle +\frac{1}{n_{w}^{2}}\sum_{k=1}^{6}\sum_{l=1}^{6}\sigma_{k}^{-2}\sigma_{l}^{-2}\langle a_{\left[k\right]}^{\left(1\right)}{\left(a_{\left[l\right]}^{\left(1\right)}\right)}^{T}\rangle \;, \end{array}$$

using $a_{\left[i\right]}$ to denote the *i*-th column of A, as defined in (14). Consequently, if i=1,2,3, then

$$a_{\left[i\right]}^{\left(1\right)}=R_{x\left[i\right]}^{\;\left(1\right)}=DR_{x\left[i\right]}^{}z_{2}{\;}^{\left(1\right)}\;,$$

otherwise, if i=4,5,6, then

$$a_{\left[i\right]}^{\left(1\right)}=R_{y\left[i-3\right]}^{\;\left(1\right)}=DR_{y\left[i-3\right]}^{}z_{3}{\;}^{\left(1\right)}\;.$$

As a result, $\langle a_{\left[i\right]}^{\left(1\right)}{\left(a_{\left[j\right]}^{\left(1\right)}\right)}^{T}\rangle$ is computed by substituting (23), (26), and (29), and their respective analogous terms for branch 1–3. For more details on the derivation of this covariance, refer to [32].

### 4.3. Covariance of Other Attitudes

The uncertainty analysis is concluded by computing the covariance matrices of $R_{2}^{I}$, $R_{3}^{I}$, and $R_{3}^{2}$. For this purpose, recall the relations in (12) and apply the first-order perturbation, similarly to (18), which yields

(33a) $$R_{2}^{I}{\;}^{\left(1\right)}=R_{1}^{I}{\;}^{\left(1\right)}R_{2}^{1}{\;}^{\left(0\right)}+R_{1}^{I}{\;}^{\left(0\right)}R_{2}^{1}{\;}^{\left(1\right)}\;,$$

(33b) $$R_{3}^{I}{\;}^{\left(1\right)}=R_{1}^{I}{\;}^{\left(1\right)}R_{3}^{1}{\;}^{\left(0\right)}+R_{1}^{I}{\;}^{\left(0\right)}R_{3}^{1}{\;}^{\left(1\right)}\;,$$

and

(33c) $$R_{3}^{2}{\;}^{\left(1\right)}={\left(R_{2}^{1}{\;}^{\left(1\right)}\right)}^{T}R_{3}^{1}{\;}^{\left(0\right)}+{\left(R_{2}^{1}{\;}^{\left(0\right)}\right)}^{T}R_{3}^{1}{\;}^{\left(1\right)}\;.$$

First, consider the covariance of $R_{2}^{I}$, which is completely analogous to the covariance of $R_{3}^{I}$, and therefore, the analysis of the latter is omitted. One method to obtain the covariance explicitly is to analyze the first-order perturbation element by element. Hence, from (33a), it follows that

(34) $$R_{2\left[i,j\right]}^{I\;\left(1\right)}={\left(R_{1\left[i\right]}^{I\;\left(1\right)}\right)}^{T}R_{2\left[j\right]}^{1\;\left(0\right)}+{\left(R_{1\left[i\right]}^{I\;\left(0\right)}\right)}^{T}R_{2\left[j\right]}^{1\;\left(1\right)}\;,$$

where $R_{2}^{I}{\;}^{\left(1\right)}\left[i,j\right]$ denotes the element of $R_{2}^{I}$ at row *i* and column *j*, and the subscript $\left[j\right]$, in $R_{2\left[j\right]}^{1\;\left(0\right)}$, denotes the *j*-th column of the respective rotation. Next, applying the definition of the covariance matrix, the respective element at the *i*-th row and *j*-th column is given as

(35) $$\Sigma_{i2\left[i,j\right]}={\langle R_{2}^{I}{\;}^{\left(1\right)}{\left(R_{2}^{I}{\;}^{\left(1\right)}\right)}^{T}\rangle }_{\left[i,j\right]}=\langle {\left({\left(R_{2}^{I}\;{\;}^{T}\right)}_{\left[i\right]}^{\left(1\right)}\right)}^{T}{\left(R_{2}^{I}\;{\;}^{T}\right)}_{\left[j\right]}^{\left(1\right)}\rangle =\sum_{k=1}^{3}\langle R_{2\left[i,k\right]}^{I\;\left(1\right)}R_{2\left[j,k\right]}^{I\;\left(1\right)}\rangle \;,$$

where the subscript $\left[i,k\right]$ denotes the element at the *i*-th row and *k*-th column. Then, substituting (34) in (35) gives

(36) $$\begin{array}{cc} \Sigma_{i2\left[i,j\right]}={\langle R_{2}^{I}{\;}^{\left(1\right)}{\left(R_{2}^{I}{\;}^{\left(1\right)}\right)}^{T}\rangle }_{\left[i,j\right]}\; & =\sum_{k=1}^{3}{\left({\left(R_{1}^{I}\;{\;}^{T}\right)}_{\left[i\right]}^{\left(0\right)}\right)}^{T}\langle R_{2\left[k\right]}^{1\;\left(1\right)}{\left(R_{2\left[k\right]}^{1\;\left(1\right)}\right)}^{T}\rangle {\left(R_{1}^{I}\;{\;}^{T}\right)}_{\left[j\right]}^{\left(0\right)}\\ & +{\left(R_{2\left[k\right]}^{1\;\left(0\right)}\right)}^{T}\langle {\left(R_{1}^{I}\;{\;}^{T}\right)}_{\left[i\right]}^{\left(1\right)}{\left(R_{2\left[k\right]}^{1\;\left(1\right)}\right)}^{T}\rangle {\left(R_{1}^{I}\;{\;}^{T}\right)}_{\left[j\right]}^{\left(0\right)}\\ & +{\left({\left(R_{1}^{I}\;{\;}^{T}\right)}_{\left[i\right]}^{\left(0\right)}\right)}^{T}\langle R_{2\left[k\right]}^{1\;\left(1\right)}{\left({\left(R_{1}^{I}\;{\;}^{T}\right)}_{\left[j\right]}^{\left(1\right)}\right)}^{T}\rangle R_{2\left[k\right]}^{1\;\left(0\right)}\\ & +{\left(R_{2\left[k\right]}^{1\;\left(0\right)}\right)}^{T}\langle {\left(R_{1}^{I}\;{\;}^{T}\right)}_{\left[i\right]}^{\left(1\right)}{\left({\left(R_{1}^{I}\;{\;}^{T}\right)}_{\left[j\right]}^{\left(1\right)}\right)}^{T}\rangle R_{2\left[k\right]}^{1\;\left(0\right)}\;. \end{array}$$

Recalling (31) and that $R_{1}^{I}\;{\;}^{T}\approx \left(I-S\left(\delta \theta_{1i}\right)\right)R_{1}^{I}\;{\;}^{T}{\;}^{\left(0\right)}$, it is concluded that $\delta \theta_{i1}=-R_{1}^{I}{\;}^{\left(0\right)}\delta \theta_{1i}$. As a result, it follows that

$$\langle {\left(R_{1}^{I}\;{\;}^{T}\right)}_{\left[i\right]}^{\left(1\right)}{\left({\left(R_{1}^{I}\;{\;}^{T}\right)}_{\left[j\right]}^{\left(1\right)}\right)}^{T}\rangle =S\left({\left(R_{1}^{I}\;{\;}^{T}\right)}_{\left[i\right]}^{\left(0\right)}\right){\left(R_{1}^{I}{\;}^{\left(0\right)}\right)}^{T}\langle \delta \theta_{i1}\delta \theta_{i1}^{T}\rangle R_{1}^{I}{\;}^{\left(0\right)}S{\left({\left(R_{1}^{I}\;{\;}^{T}\right)}_{\left[i\right]}^{\left(0\right)}\right)}^{T}$$

with $\langle \delta \theta_{i1}\delta \theta_{i1}^{T}\rangle$ given in (32). Moreover, the expected value in the first term of (36) is obtained from the linearization (20), which results in

(37) $$\langle R_{2\left[k\right]}^{1\;\left(1\right)}{\left(R_{2\left[k\right]}^{1\;\left(1\right)}\right)}^{T}\rangle =DR_{2\left[k\right]}^{1}\langle z_{2}{\;}^{\left(1\right)}{\left(z_{2}{\;}^{\left(1\right)}\right)}^{T}\rangle {\left(DR_{2\left[k\right]}^{1}\right)}^{T}\;,$$

where $\langle z_{2}{\;}^{\left(1\right)}{\left(z_{2}{\;}^{\left(1\right)}\right)}^{T}\rangle$ is given in (26). Lastly, the expected value of the second term of (36), which is analogous to the expected value in the third term, is expressed as

$$\langle {\left(R_{1}^{I}\;{\;}^{T}\right)}_{\left[k\right]}^{\left(1\right)}{\left(R_{2\left[j\right]}^{1\;\left(1\right)}\right)}^{T}\rangle =S\left({\left(R_{1}^{I}\;{\;}^{T}\right)}_{\left[i\right]}^{\left(0\right)}\right){\left(R_{1}^{I}{\;}^{\left(0\right)}\right)}^{T}\langle \delta \theta_{i1}{\left(z_{2}{\;}^{\left(1\right)}\right)}^{T}\rangle {\left(DR_{2\left[j\right]}^{1}\right)}^{T}\;.$$

From the definition of $\delta \theta_{i1}$ given in [32], it follows that

$$\langle \delta \theta_{i1}{\left(z_{2}{\;}^{\left(1\right)}\right)}^{T}\rangle =H^{-1}\sum_{m=1}^{6}-\sigma_{m}^{-2}S\left(R_{1}^{I}{\;}^{\left(0\right)}b_{\left[m\right]}\right)\left(\langle a_{\left[m\right]}{\left(z_{2}{\;}^{\left(1\right)}\right)}^{T}\rangle -\sum_{n=1}^{6}\sigma_{n}^{-2}\langle a_{\left[n\right]}{\left(z_{2}{\;}^{\left(1\right)}\right)}^{T}\rangle \right)\;,$$

where $a_{\left[m\right]}=R_{x\left[m\right]}^{\;\left(1\right)}$ if m=1,2,3 or $a_{\left[m\right]}=R_{y\left[m-3\right]}^{\;\left(1\right)}$ if m=4,5,6, and therefore, from (22),

$$\langle a_{\left[m\right]}{\left(z_{2}{\;}^{\left(1\right)}\right)}^{T}\rangle =DR_{x\left[m\right]}^{}\langle z_{2}{\;}^{\left(1\right)}{\left(z_{2}{\;}^{\left(1\right)}\right)}^{T}\rangle \;\mathrm{or}\;\langle a_{\left[m\right]}{\left(z_{2}{\;}^{\left(1\right)}\right)}^{T}\rangle =DR_{y\left[m-3\right]}^{}\langle z_{3}{\;}^{\left(1\right)}{\left(z_{2}{\;}^{\left(1\right)}\right)}^{T}\rangle \;,$$

respectively.

Using the same train of thought for the covariance of $R_{3}^{2}$, the perturbation in (33c) is rewritten as

(38) $$R_{3\left[i,j\right]}^{2\;\left(1\right)}={\left(R_{2\left[i\right]}^{1\;\left(1\right)}\right)}^{T}R_{3\left[j\right]}^{1\;\left(0\right)}+{\left(R_{2\left[i\right]}^{1\;\left(0\right)}\right)}^{T}R_{3\left[j\right]}^{1\;\left(1\right)}\;,$$

which, applying the definition of covariance matrix, gives

$$\Sigma_{23\left[i,j\right]}={\langle R_{3}^{2}{\;}^{\left(1\right)}{\left(R_{3}^{2}{\;}^{\left(1\right)}\right)}^{T}\rangle }_{\left[i,j\right]}=\sum_{k=1}^{3}\langle R_{3\left[i,k\right]}^{2\;\left(1\right)}R_{3\left[j,k\right]}^{2\;\left(1\right)}\rangle \;.$$

Then, from (38), it follows that

(39) $$\begin{array}{cc} \Sigma_{23\left[i,j\right]}= & \sum_{k=1}^{3}{\left(R_{3\left[k\right]}^{1\;\left(0\right)}\right)}^{T}\langle R_{2\left[i\right]}^{1\;\left(1\right)}{\left(R_{2\left[j\right]}^{1\;\left(1\right)}\right)}^{T}\rangle R_{3\left[k\right]}^{1\;\left(0\right)}\\ & +{\left(R_{2\left[i\right]}^{1\;\left(0\right)}\right)}^{T}\langle R_{3\left[k\right]}^{1\;\left(1\right)}{\left(R_{3\left[k\right]}^{1\;\left(1\right)}\right)}^{T}\rangle R_{2\left[j\right]}^{1\;\left(0\right)}\\ & +{\left(R_{2\left[i\right]}^{1\;\left(0\right)}\right)}^{T}\langle R_{3\left[k\right]}^{1\;\left(1\right)}{\left(R_{2\left[j\right]}^{1\;\left(1\right)}\right)}^{T}\rangle R_{3\left[k\right]}^{1\;\left(0\right)}\\ & +{\left(R_{3\left[k\right]}^{1\;\left(0\right)}\right)}^{T}\langle R_{2\left[i\right]}^{1\;\left(1\right)}{\left(R_{3\left[k\right]}^{1\;\left(1\right)}\right)}^{T}\rangle R_{2\left[j\right]}^{1\;\left(0\right)}\;. \end{array}$$

The explicit expected values in (39) are given next. First, $\langle R_{2\left[i\right]}^{1\;\left(1\right)}{\left(R_{2\left[j\right]}^{1\;\left(1\right)}\right)}^{T}\rangle$ is given in (37). Secondly, from the analogous term of (20), it follows that

$$\langle R_{3\left[k\right]}^{1\;\left(1\right)}{\left(R_{3\left[k\right]}^{1\;\left(1\right)}\right)}^{T}\rangle =DR_{3\left[k\right]}^{1}\langle z_{3}{\;}^{\left(1\right)}{\left(z_{3}{\;}^{\left(1\right)}\right)}^{T}\rangle {\left(DR_{3\left[k\right]}^{1}\right)}^{T}\;,$$

with $\langle z_{3}{\;}^{\left(1\right)}{\left(z_{3}{\;}^{\left(1\right)}\right)}^{T}\rangle$ being the analogous term of (26). Finally, the cross expected value in the third term, and analogously in the fourth term, is given as

$$\langle R_{3\left[k\right]}^{1\;\left(1\right)}{\left(R_{2\left[j\right]}^{1\;\left(1\right)}\right)}^{T}\rangle =DR_{3\left[k\right]}^{1}\langle z_{3}{\;}^{\left(1\right)}{\left(z_{2}{\;}^{\left(1\right)}\right)}^{T}\rangle {\left(DR_{2\left[j\right]}^{1}\right)}^{T}\;,$$

with $\langle z_{3}{\;}^{\left(1\right)}{\left(z_{2}{\;}^{\left(1\right)}\right)}^{T}\rangle$ given in (29).

### 4.4. Sensitivity in Special Configurations

The uncertainty analysis is invalid in the cases where there is more than one solution to the attitude problem. However, it is important to analyze and understand the expected noise in the neighborhood of such configurations. In the following analysis, consider branch 1–2.

**Figure 2:**
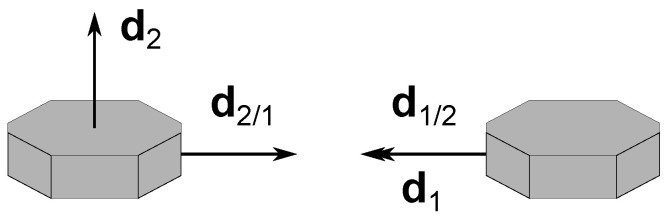
Example of a degenerate branch 1–2.

Near degenerate configurations, as with the example depicted in Figure 2, where there are at least two independent measurements aligned with each other, the error increases because such alignment results in the loss of attitude information. In the limit, both $c_{s12}$ and $c_{c12}$ tend to zero and the uncertainty becomes infinitely large, as predicted in (A1).

**Figure 3:**
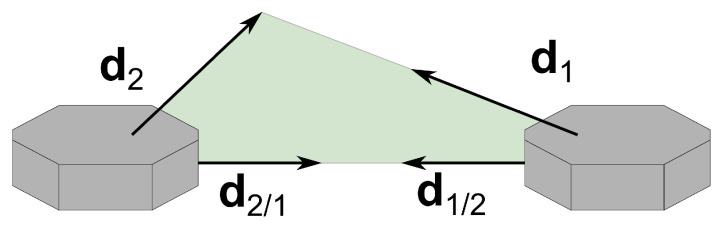
Example of a coplanar branch 1–2.

Near coplanar, but not degenerate, configurations, as with the example depicted in Figure 3, where the branch measurements denoted in the same frame are coplanar, the error increases for the same reason as in the degenerate cases, i.e., loss of information. In such a case, the branch has a unique solution in the absence of noise, but the uncertainty becomes increasingly larger. Such errors are predicted in (A1), because, by definition, from (6a), $c_{s12}^{2}+c_{c12}^{2}\approx c_{p12}^{2}$ in the neighborhood of such configurations.

**Figure 4:**
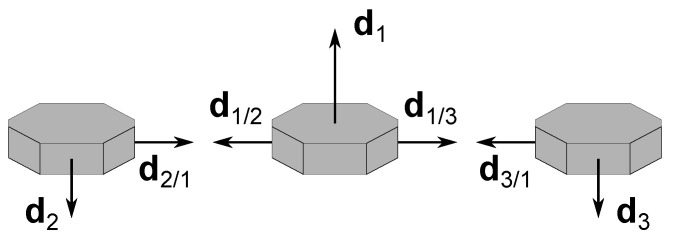
Example of an ambiguous formation.

The ambiguous, but not degenerate or coplanar, configurations, as the in example in Figure 4, do not affect the theoretical covariance in their neighborhood. In their vicinity, the errors may greatly increase in practice because the solution may jump to the incorrect candidate of the branch.

These special configurations are in a zero measure subset of the complete configuration set, which was shown in [29]. Consequently, these configurations have zero probability in practice, even though they can affect the accuracy of the solution in configurations in their neighborhood, as will be shown in the simulations.

## 5. Simulations

The covariance matrices obtained in the prior section are validated by comparing the results of the numerical implementation with the theoretical values predicted in such expressions. The goal is to show that the theoretical uncertainty is close to the numerical values and that the higher-order perturbations are not significant. For this purpose, Monte Carlo simulations are carried out. First, a series of ground truth values is selected by setting the initial configuration and the maneuver executed by the vehicles. Then, noise is added to the measurements by sampling from their respective measurement model probability distribution and the attitude estimates are obtained applying the solution described in Section 3. The experience is repeated multiple times, 1000 times to be precise, which allows the computation of the standard deviation for each of the configurations considered. Finally, the theoretical covariance is computed for each of the configurations and the respective theoretical standard deviation is compared with the standard deviation obtained numerically, by plotting both in the same axis. Three different experiences are carried out such that the predictions near each of the special configurations, as defined in [29] and whose examples are depicted in Figure 2, Figure 3 and Figure 4, are tested. The first experience contains a degenerate case, the second experience contains a coplanar case, and the third experience contains an ambiguous configuration.

### 5.1. Models

In this section, two models are described. Firstly, the measurement model is used for all sensors of the formation, which represents a focal plane array. The second is the motion model of each vehicle, which follows the rigid-body dynamics, and is required exclusively to generate the ground truth of the simulation.

#### 5.1.1. Focal Plane Array Model

The measurement model is based on a wide FOV focal plane detector [19], which can sense the direction towards a given signal. It is the same model used in the formations considered in [27,28].

Denote the image-space observation by the vector $m\equiv {\left[\chi \;\psi \right]}^{T}$. Then, the measurement model is given by $\tilde{m}=m+n$ (the image-space frame is the 2D coordinate system of the sensor, whereas the object-space frame is the vehicle body coordinate system), where $\tilde{m}$ is the measurement and n is the random noise. The noise model describing the uncertainty of the image-space observations is supposed to follow a zero mean Gaussian distribution, $n∼N\left(0,\Sigma_{F}\right)$, with the covariance of the focal plane given by [18]

(40) $$\Sigma_{F}=\frac{\sigma^{2}}{1+d\left(\chi^{2}+\psi^{2}\right)}\left[\begin{array}{cc} {\left(1+d\chi^{2}\right)}^{2} & {\left(d\chi \psi \right)}^{2}\\ {\left(d\chi \psi \right)}^{2} & {\left(1+d\psi^{2}\right)}^{2} \end{array}\right]\;,$$

where $\sigma_{}^{2}$ is the variance of the measurement errors associated with χ and ψ, and *d* is a parameter on the order of 1.

The focal length is assumed to be unitary and the sensor boresight is assumed to be the z-axis. Hence, the measurement vector in the object space and sensor frame is given as

(41) $$\;{\;}_{}^{S}\;d_{}=\frac{1}{\sqrt{1+\chi^{2}+\psi^{2}}}{\left[\begin{array}{ccc} \chi & \psi & 1 \end{array}\right]}^{T}\;.$$

Consequently, the covariance of the sensor, at the sensor frame, is given as [19]

(42) $$\Sigma_{S}=L\;\Sigma_{F}\;L^{T}\;,$$

where L is the Jacobian of the relation between the sensor and focal coordinates, which is given as

$$L=\frac{\partial \;\;{\;}_{}^{S}\;d_{}}{\partial m}=\frac{1}{\sqrt{1+\chi^{2}+\psi^{2}}}{\left[\begin{array}{ccc} 1 & 0 & 0\\ 0 & 1 & 0 \end{array}\right]}^{T}+\frac{1}{1+\chi^{2}+\psi^{2}}\;{\;}_{}^{S}\;d_{}\left[\begin{array}{cc} \chi & \psi \end{array}\right]\;.$$

Furthermore, it is assumed that there are sensors aligned with each body-fixed axis direction and the measurement is made by the sensor whose value of $\tilde{m}$ is closest to 0. Hence, the six possible transformations between the body-fixed frame and the sensor frame are given by

(43a) $$R_{B}^{S_{1}}=\left[\begin{array}{ccc} 0 & 0 & -1\\ 0 & 1 & 0\\ 1 & 0 & 0 \end{array}\right]\;,R_{B}^{S_{2}}=\left[\begin{array}{ccc} 1 & 0 & 0\\ 0 & 0 & -1\\ 0 & 1 & 0 \end{array}\right]\;,\;\mathrm{and}\;R_{B}^{S_{3}}=\left[\begin{array}{ccc} 1 & 0 & 0\\ 0 & 1 & 0\\ 0 & 0 & 1 \end{array}\right]\;,$$

respectively, when the measurement component with the maximum absolute value is positive and is either 1, 2, or 3. If the maximum absolute value component is negative, then the analogous transformations are given by

(43b) $$R_{B}^{S_{-1}}=\left[\begin{array}{ccc} 0 & 0 & 1\\ 0 & 1 & 0\\ -1 & 0 & 0 \end{array}\right]\;,R_{B}^{S_{-2}}=\left[\begin{array}{ccc} 1 & 0 & 0\\ 0 & 0 & 1\\ 0 & -1 & 0 \end{array}\right]\;,\;\mathrm{and}\;R_{B}^{S_{-3}}=\left[\begin{array}{ccc} -1 & 0 & 0\\ 0 & 1 & 0\\ 0 & 0 & -1 \end{array}\right]\;.$$

#### 5.1.2. Motion Model

The simulation ground truth considers the rigid-body dynamics such that the attitude varies with time despite the estimation method being algebraic. Such a feature enables the assessment of the uncertainty obtained in Section 4 for different attitude values. Therefore, the motion model of the *j*-th vehicle is given by the attitude kinematics and the torque-free rigid-body dynamics, respectively, expressed as

(44) R˙jI=RjISωj,

and

(45) $${\dot{\omega }}_{j}=-J_{j}^{-1}S\left(\omega_{j}\right)J_{j}\omega_{j}\;,$$

where ${\dot{\omega }}_{j}$ denotes the angular velocity of the *j*-th vehicle and $J_{j}$ denotes the matrix of the moment of inertia given in $\mathrm{kg}\cdot m^{2}$.

### 5.2. Setup

#### 5.2.1. Initial Configuration

**Figure 5:**
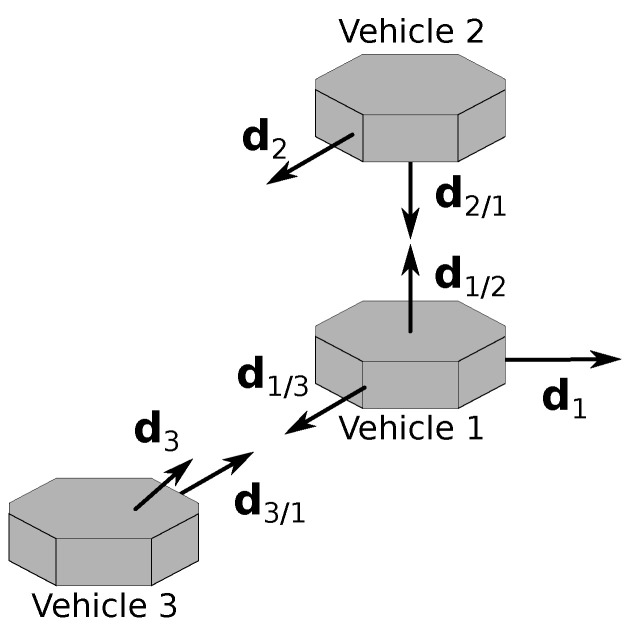
Initial configuration for the simulation.

The initial configuration, which is illustrated in Figure 5, is identical for all three experiments and is described by the values of the inertial attitudes and measurements. Thus, the attitudes are initially given by $R_{1}^{I}=R_{2}^{I}=R_{3}^{I}=I$, whereas the initial values of the LOS measurements at the inertial frame are given by

(46a) Id1/2=001T.

and

(46b) $$\;{\;}^{I}\;d_{1/3}=\left[\begin{array}{ccc} 1 & 0 & 0 \end{array}\right]{}^{T}\;,$$

and, finally, the initial inertial references are given by

(47a) $$\;{\;}^{I}\;d_{1}={\left[\begin{array}{ccc} 0 & 1 & 0 \end{array}\right]}^{T}\;,$$

(47b) $$\;{\;}^{I}\;d_{2}={\left[\begin{array}{ccc} 1 & 0 & 0 \end{array}\right]}^{T}\;,$$

and

(47c) $$\;{\;}^{I}\;d_{3}={\left[\begin{array}{ccc} \sqrt{3}/3 & \sqrt{3}/3 & \sqrt{3}/3 \end{array}\right]}^{T}\;.$$

These values define the initial configuration because all relative attitudes can be computed from the product of two inertial attitudes and the values of the measurements represented in different frames are obtained by applying one of the rotations.

#### 5.2.2. Motion Parameters

The rigid-body dynamics of all three vehicles are characterized by the moment of inertia and the initial angular velocity. In this simulation, the moment of inertia is the same for all three vehicles and is given by $J_{1}=J_{2}=J_{3}=\mathrm{diag}\left(70,70,60\right)$, which corresponds to a cylindrical vehicle with 120 kg, 2 m of height, and the radius equal to 1 m, and the initial angular velocities, in rad/s, are given by $\omega_{1}=\left[\begin{array}{ccc} 0.1 & 0 & 0 \end{array}\right]{}^{T}$, $\omega_{2}=\left[\begin{array}{ccc} 0 & 0.1 & 0 \end{array}\right]{}^{T}$, and $\omega_{3}=\left[\begin{array}{ccc} 0 & 0 & 0.1 \end{array}\right]{}^{T}$.

#### 5.2.3. Maneuvers

Throughout each experience, the configuration of the vehicles is changed by a maneuver described by a rotation of $\;{\;}^{I}\;d_{1/3}$, which affects the value of $d_{1/3}$ and $d_{3/1}$. All other measurements are constant in the inertial coordinate frame.

The first experience considers that the value of $\;{\;}^{I}\;d_{1/3}$ at the time instant *t* is given, recalling (1), by

(48a) $$\;{\;}^{I}\;d_{1/3}\left(t\right)=R\left(\pi \frac{t}{100},{\left[\begin{array}{ccc} 0 & 0 & 1 \end{array}\right]}^{T}\right)\;{\;}^{I}\;d_{1/3}\left(0\right)\;,$$

where $\;{\;}^{I}\;d_{1/3}\left(0\right)$ denotes the initial value defined in (46b). Instead, the second experience considers that the value of $\;{\;}^{I}\;d_{1/3}$ at the time instant *t* is given by

(48b) $$\;{\;}^{I}\;d_{1/3}\left(t\right)=R\left(-\pi \frac{t}{200},{\left[\begin{array}{ccc} 0 & 1 & 0 \end{array}\right]}^{T}\right)\;{\;}^{I}\;d_{1/3}\left(0\right)\;.$$

Finally, the third experience assumes that the value of $\;{\;}^{I}\;d_{1/3}$ at the time instant *t* is given by

(48c) $$\;{\;}^{I}\;d_{1/3}\left(t\right)=R\left(\pi \frac{t}{200},{\left[\begin{array}{ccc} 0 & 1 & 0 \end{array}\right]}^{T}\right)\;{\;}^{I}\;d_{1/3}\left(0\right)\;.$$

All maneuvers take 100 s. The first rotates a total angle of π, the second a total angle of $-\frac{\pi }{2}$, and the third a total angle of $\frac{\pi }{2}$.

#### 5.2.4. Simulation Procedure

All experiments consider a set of 1000 Monte Carlo trials that run for 100 s with a sampling frequency of 10 Hz, which means that a measurement is made each 0.1 s. The initial configuration and respective maneuver are those described previously. Moreover, all sensors are characterized by a standard deviation of $\sigma_{}=17\times 10^{-6}$ rad.

In each experience, the ground truth values of the attitudes and angular velocities are computed beforehand by solving the initial value problem given by (44) and (45). Then, considering the initial values in the inertial frame given in (46) and (47), the ground truth measurements are computed recalling (48a), (48b), or (48c), respectively, for the first, the second, or the third experience, while all other measurements in the inertial frame are constant. The measurements represented in the body-fixed frames are computed using the attitudes of the respective time instant. After computing the ground truth, the simulation trials begin. Each trial consists in two tasks: first adding noise added to the ground truth measurements and then computing the attitude estimates. There are 1000 trials in each experience, which means that the attitude is estimated from noisy measurements 1000 times for each sampling instant.

More specifically, in each trial and at each sampling instant, noise is added to each of the measurements following the measurement model described in Section 5.1.1. For this, the true measurements are transformed into the appropriate sensor frame, using the correct transformation in (43). Then, the focal coordinates are obtained from (41). Next, the covariance in the sensor frame is computed from (40) and (42). Such covariance is used to sample the noise in the sensor frame, which is then added to the respective measurement. The perturbed measurement in the body-fixed frame is obtained, after applying the appropriate reverse transformation in (43). Finally, the attitude estimates are computed applying the method summarized in Section 3, and the respective error rotation vectors, considering $R_{2}^{1}$, $R_{3}^{1}$, and $R_{1}^{I}$, are obtained from

$$\delta \theta_{12}=S^{{}^{-1}}\;\;\left(R_{2}^{1}{\;}^{\left(0\right)}R_{2}^{1}\;{\;}^{T}-I\right)\;,$$

$$\delta \theta_{13}=S^{{}^{-1}}\;\;\left(R_{3}^{1}{\;}^{\left(0\right)}R_{3}^{1}\;{\;}^{T}-I\right)\;,$$

and

$$\delta \theta_{i1}=S^{{}^{-1}}\;\;\left(R_{1}^{I}{\;}^{\left(0\right)}R_{1}^{I}\;{\;}^{T}-I\right)\;.$$

After all trials have finished, the element-wise standard deviation is obtained for each time instant.

The theoretical expected values of $\delta \theta_{12}$ and $\delta \theta_{i1}$ are obtained from the square root of the diagonal elements of the covariance matrices, respectively, given in (27) and (32). Both are computed for each time instant using the ground truth values of the simulation. Moreover, the theoretical value of $\delta \theta_{13}$ is similarly obtained from the analogous term of (27).

### 5.3. Results

The results for each experience are given as the theoretical and numerical values for $\delta \theta_{12}$, $\delta \theta_{13}$, and $\delta \theta_{i1}$, since these are the attitudes which are directly computed from the solution in Section 3.

**Figure 6:**
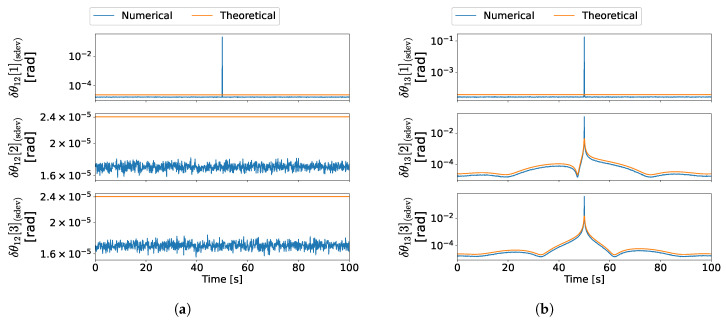

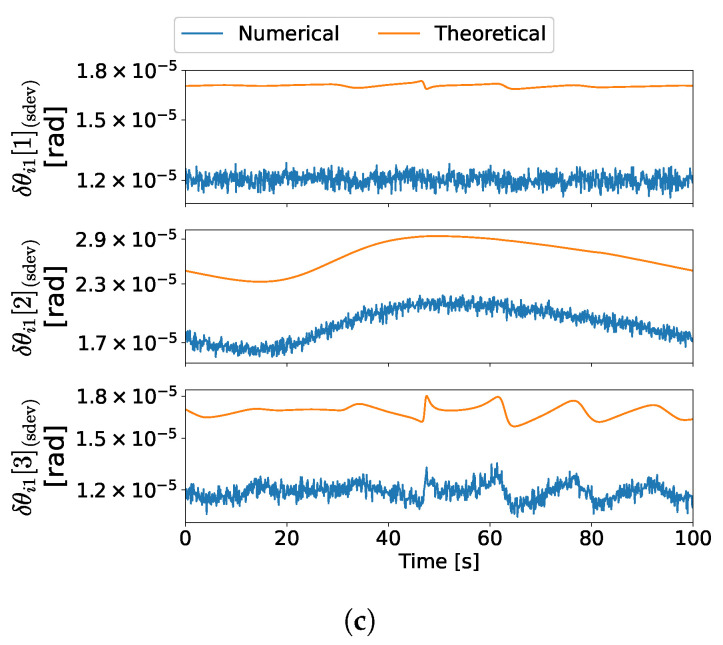
Attitude error standard deviations in experience 1. (**a**) $R_{2}^{1}$ error standard deviation in experience 1, (**b**) $R_{3}^{1}$ error standard deviation in experience 1, (**c**) $R_{1}^{I}$ error standard deviation in experience 1.

**Figure 7:**
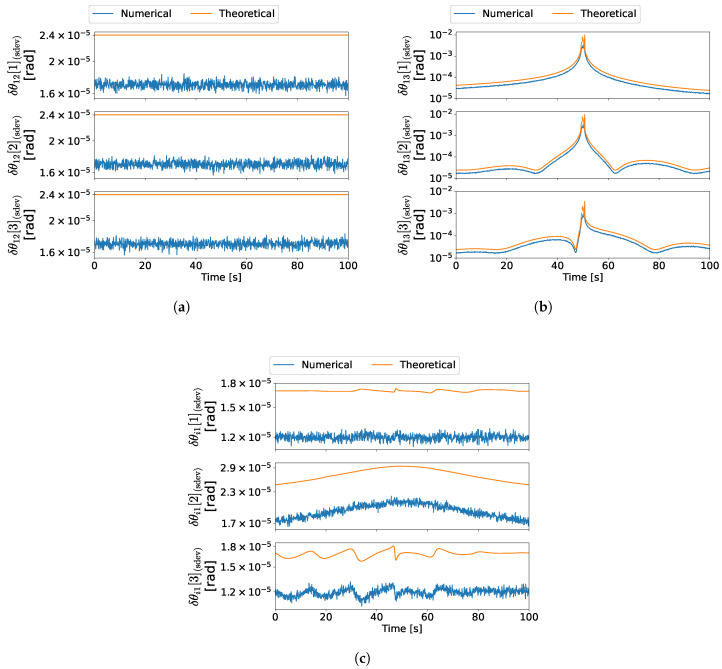
Attitude error standard deviations in experience 2. (**a**) $R_{2}^{1}$ error standard deviation in experience 2, (**b**) $R_{3}^{1}$ error standard deviation in experience 2, (**c**) $R_{1}^{I}$ error standard deviation in experience 2.

**Figure 8:**
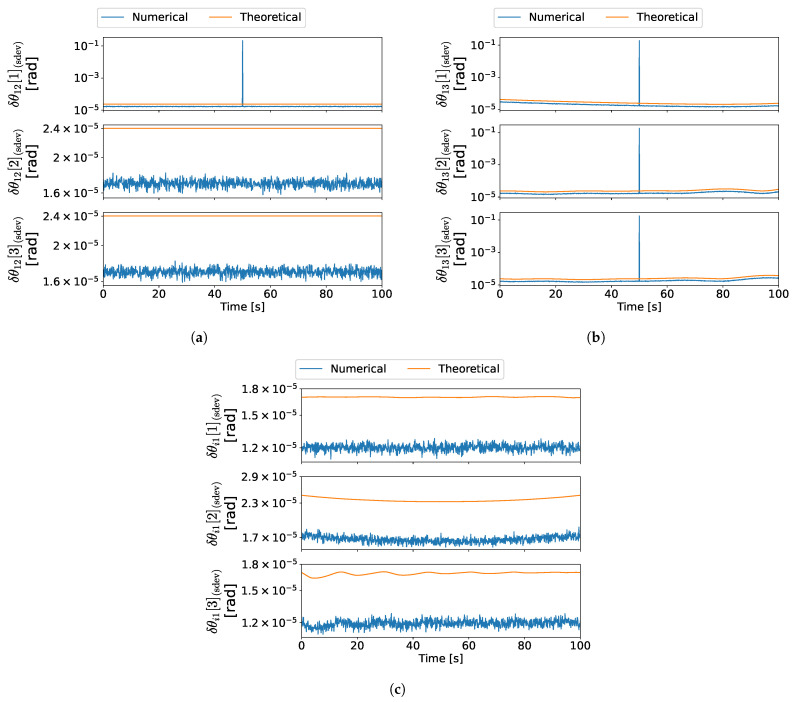
Attitude error standard deviations in experience 3. (**a**) $R_{2}^{1}$ error standard deviation in experience 3, (**b**) $R_{3}^{1}$ error standard deviation in experience 3, (**c**) $R_{1}^{I}$ error standard deviation in experience 3.

For the first experience, which corresponds to the maneuver in (48a), the results are depicted in Figure 6a–c. Moreover, the results for the second experience are depicted in Figure 7a–c, which correspond to the maneuver in (48b). Lastly, the results for the third experience are depicted in Figure 8a–c, which correspond to the maneuver in (48c).

#### Results Analysis

**Figure 9:**
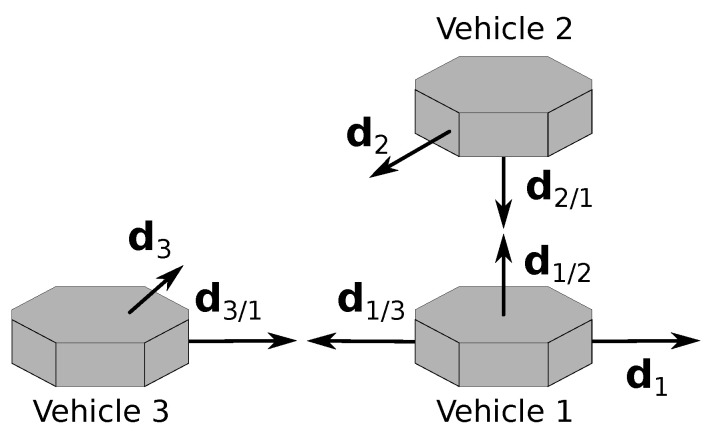
Ambiguous and degenerate configuration at 50 s of experience 1.

In the first experience, the configuration at 50 s is both degenerate for branch 1–3 and ambiguous regarding the complete formation, which is depicted in Figure 9. Since $d_{1/3}=-d_{1}$, then the measurement set also satisfies the ambiguous conditions established in [29]. Therefore, close to this time instant, the information provided by the measurements becomes gradually insufficient to determine all three axes of the relative attitude between vehicles 1 and 3. Moreover, in the vicinity of the 50 s, the information available is ambiguous; thus, the solution alternates between two distinct attitude sets. Hence, close to such time, the error increases gradually for $R_{3}^{1}$, which is predicted theoretically, as observed in Figure 6b. In the case of $R_{2}^{1}$, however, the sudden increase in the numerical error at the 50 s time instant is not predicted by the theoretical covariance, as seen in Figure 6a, because the respective expression is not valid for ambiguous configurations. Nonetheless, such configurations were previously characterized in [29] and can be classified prior to obtaining the attitude. Lastly, it is evident that the theoretical errors are close to the numerical errors for the configurations where the uncertainty characterization is valid.

In the second experience, there is a coplanar configuration at 50 s, which is analogous to the depiction in Figure 3. Similarly to the degenerate configuration in the first experience, the information provided by the measurements becomes gradually insufficient to determine all three axes of $R_{3}^{1}$. Hence, it is expected that both the theoretical predictions and the numerical results are analogous. As seen by comparing Figure 7b with Figure 6b, the results are indeed similar to those obtained in the first experience. Furthermore, the theoretical errors are close to the numerical errors for all attitudes except for the configurations where the analysis is not valid close to the 50 s time instant.

In the third experience, there is an ambiguous configuration at 50 s, which is analogous to the configuration in Figure 4. Therefore, the uncertainty analysis is invalid near this time instant. It is evident from Figure 8a,c that, near such a configuration, the numerical errors increase suddenly, as a result of the solution alternating between two distinct attitudes [29].

Lastly, as expected, the Procrustes optimization provides an improvement to the solution since it gives a good estimate for $R_{1}^{I}$ even when the uncertainty for one of the candidates is high. Figure 6 and Figure 7 are an example of such precision improvement, since the errors of $R_{1}^{I}$ are kept low as the errors of $R_{3}^{1}$ increase.

In conclusion, the results show that the first-order perturbations give a good approximation of the numerical errors in configurations which are not too close to one of the special configurations described in [29]. Furthermore, the gradual loss of precision near degenerate configurations is also predicted by the theoretical covariance.

## 6. Discussion

Attitude estimation is an essential part of the navigation, guidance, and control of any autonomous system. Formations of autonomous vehicles are especially sensitive to the accuracy of the attitude estimates in order to reach their goals safely. Furthermore, the design of these systems and their mission is often subject to constraints, and, thus, the attitude estimation must be accurate even in such conditions. The attitude estimation problem studied in this document considers three vehicles in a constrained formation.

The uncertainty analysis of an estimation problem enables the characterization of its precision in different conditions. It is essential for the integration of such estimates in sensor fusion and for real-world application decisions. The analysis given in this document characterizes the accuracy of the attitude for the three-vehicle constrained formation in almost all configurations. The exceptions are the special cases where information is lost or where the solution is not unique.

After briefly summarizing the problem and solution, the theoretical covariance matrices associated with the errors of each attitude were computed resorting to first-order perturbations in the measurements. Then, such results were validated with three different Monte Carlo simulations, where the theoretical prediction was compared with the statistical errors of multiple numerical implementations of the solution. It was shown that the theoretical predictions were consistent with the numerical results, even in the neighborhood of the special configurations, which validates the uncertainty analysis proposed in this paper.

## Data Availability

Not applicable.

## Abbreviations

The following abbreviations are used in this manuscript:

EKF	Extended Kalman filter
ESOQ	Estimator of the Optimal Quaternion
ESOQ2	Second Estimator of the Optimal Quaternion
FLAE	Fast Linear Quaternion Attitude Estimator
FOAM	Fast Optimal Attitude Matrix
FOV	Field-of-view
LOS	Line-of-sight
QUEST	Quaternion Estimator
SVD	Singular value decomposition
TRIAD	Tri-Axial Attitude Determination

## Appendix A. Partial Derivatives

This appendix contains all the expressions for the partial derivatives required in the construction of the Jacobian matrices used in the linearization. For the remainder of the section, let $e_{\left[j\right]}$ denote the *j*-th standard unit vector of the Euclidean space, respectively, defined as $e_{\left[1\right]}:=\left[\begin{array}{ccc} 1 & 0 & 0 \end{array}\right]$, $e_{\left[2\right]}:=\left[\begin{array}{ccc} 0 & 1 & 0 \end{array}\right]$, and $e_{\left[3\right]}:=\left[\begin{array}{ccc} 0 & 0 & 1 \end{array}\right]$.

### Appendix A.1. Partial Derivative of $R_{2}^{1}$

According to (4), the relative attitude varies with $n_{1}$, $n_{2}$, and $\theta_{2}$. Hence, from (1), it follows that

$$\frac{\partial R_{2}^{1}}{\partial n_{1\left[j\right]}}=2R\left(\theta_{2},n_{2}\right)\left(e_{\left[j\right]}n_{1}^{T}+n_{1}e_{\left[j\right]}^{T}\right)\;,$$

$$\frac{\partial R_{2}^{1}}{\partial n_{2\left[j\right]}}=\left[\left(1-\cos\theta_{2}\right)\left(e_{\left[j\right]}n_{2}^{T}+n_{2}e_{\left[j\right]}^{T}\right)-\sin\theta_{2}S\left(e_{\left[j\right]}\right)\right]R\left(\theta_{1},n_{1}\right)\;,$$

and

$$\frac{\partial R_{2}^{1}}{\partial \theta_{2}}=\left[\sin\theta_{2}\left(n_{2}n_{2}^{T}-I\right)-\cos\theta_{2}S\left(n_{2}\right)\right]R\left(\theta_{1},n_{1}\right)\;.$$

### Appendix A.2. Partial Derivatives of $n_{1}$

According to (5b), $n_{1}$ varies directly with $d_{1/2}$ and $d_{2/1}$. Hence, it follows that

∂n1∂d1/2j=n1n1T−Id2/1−d1/2ejifd2/1≠d1/2I−n1n1TSd1/2d1Sejd1otherwise.

and

$$\frac{\partial n_{1}}{\partial d_{2/1\left[j\right]}}=\left\{\begin{array}{cc} \frac{\left[I-n_{1}n_{1}^{T}\right]}{∥d_{2/1}-d_{1/2}∥}e_{\left[j\right]}\; & \mathrm{if}\;d_{2/1}\neq d_{1/2}\\ \;0\; & \mathrm{otherwise} \end{array}\right.\;.$$

### Appendix A.3. Partial Derivatives of $n_{2}$

Since $n_{2}=-d_{1/2}$, it follows that $\frac{\partial n_{2}}{\partial d_{1/2\left[j\right]}}=-e_{\left[j\right]}$.

### Appendix A.4. Partial Derivatives of $\theta_{2}$

According to (6a), $\theta_{2}$ is a function of the scalar coefficients defined in (7). Hence, it follows that

(A1a) $$\frac{\partial \theta_{2}}{\partial c_{s12}}=\frac{1}{c_{s12}^{2}+c_{c12}^{2}}\left(c_{c12}+\sigma_{12}\frac{c_{s12}c_{p12}}{\sqrt{c_{s12}^{2}+c_{c12}^{2}-c_{p12}^{2}}}\right)\;,$$

(A1b) $$\frac{\partial \theta_{2}}{\partial c_{c12}}=\frac{1}{c_{s12}^{2}+c_{c12}^{2}}\left(-c_{s12}+\sigma_{12}\frac{c_{c12}c_{p12}}{\sqrt{c_{s12}^{2}+c_{c12}^{2}-c_{p12}^{2}}}\right)\;,$$

and

(A1c) $$\frac{\partial \theta_{2}}{\partial c_{p12}}=-\frac{\sigma }{\sqrt{c_{s12}^{2}+c_{c12}^{2}-c_{p12}^{2}}}\;.$$

### Appendix A.5. Partial Derivatives of $c_{s12}$

Recalling (7) and (8), it follows that

$$\frac{\partial c_{s12}}{\partial d_{1\left[j\right]}}=e_{\left[j\right]}^{T}S\left(-d_{1/2}\right)d_{2}^{⋆}\;,$$

$$\frac{\partial c_{s12}}{\partial d_{1/2\left[j\right]}}=d_{1}^{T}S\left(-e_{\left[j\right]}\right)d_{2}^{⋆}+2d_{1}^{T}S\left(-d_{1/2}\right)\left[\frac{\partial n_{1}}{\partial d_{1/2\left[j\right]}}n_{1}^{T}+n_{1}{\frac{\partial n_{1}}{\partial d_{1/2\left[j\right]}}}^{T}\right]d_{2}\;,$$

$$\frac{\partial c_{s12}}{\partial d_{2/1\left[j\right]}}=2d_{1}^{T}S\left(-d_{1/2}\right)\left[\frac{\partial n_{1}}{\partial d_{2/1\left[j\right]}}n_{1}^{T}+n_{1}{\frac{\partial n_{1}}{\partial d_{2/1\left[j\right]}}}^{T}\right]d_{2}\;,$$

and

$$\frac{\partial c_{s12}}{\partial d_{2\left[j\right]}}=d_{1}^{T}S\left(-d_{1/2}\right)R\left(\theta_{1},n_{1}\right)e_{\left[j\right]}\;.$$

### Appendix A.6. Partial Derivatives of $c_{c12}$

Recalling (7) and (8), it follows that

$$\frac{\partial c_{c12}}{\partial d_{1\left[j\right]}}=e_{\left[j\right]}^{T}S\left(d_{1/2}\right)S\left(d_{1/2}\right)d_{2}^{⋆}\;,$$

$$\begin{array}{c} \frac{\partial c_{c12}}{\partial d_{1/2\left[j\right]}}=d_{1}^{T}S\left(e_{\left[j\right]}\right)S\left(d_{1/2}\right)d_{2}^{⋆}+d_{1}^{T}S\left(d_{1/2}\right)S\left(e_{\left[j\right]}\right)d_{2}^{⋆}\;\;\;\;\;\;\;\;\;\;\;\\ \;\;\;\;\;\;\;\;\;\;\;+2d_{1}^{T}S\left(d_{1/2}\right)S\left(d_{1/2}\right)\left[\frac{\partial n_{1}}{\partial d_{1/2\left[j\right]}}n_{1}^{T}+n_{1}{\frac{\partial n_{1}}{\partial d_{1/2\left[j\right]}}}^{T}\right]d_{2}\;, \end{array}$$

$$\frac{\partial c_{c12}}{\partial d_{2/1\left[j\right]}}=2d_{1}^{T}S\left(d_{1/2}\right)S\left(d_{1/2}\right)\left[\frac{\partial n_{1}}{\partial d_{2/1\left[j\right]}}n_{1}^{T}+n_{1}{\frac{\partial n_{1}}{\partial d_{2/1\left[j\right]}}}^{T}\right]d_{2}\;,$$

and

$$\frac{\partial c_{c12}}{\partial d_{2\left[j\right]}}=d_{1}^{T}S\left(d_{1/2}\right)S\left(d_{1/2}\right)R\left(\theta_{1},n_{1}\right)e_{\left[j\right]}\;.$$

### Appendix A.7. Partial Derivatives of $c_{p12}$

Recalling (7) and (8), it follows that

$$\frac{\partial c_{p12}}{\partial d_{1\left[j\right]}}=e_{\left[j\right]}^{T}d_{1/2}d_{1/2}^{T}d_{2}^{⋆}\;,$$

$$\frac{\partial c_{p12}}{\partial d_{1/2\left[j\right]}}=d_{1}^{T}e_{\left[j\right]}d_{1/2}^{T}d_{2}^{⋆}+d_{1}^{T}d_{1/2}e_{\left[j\right]}^{T}d_{2}^{⋆}+2d_{1}^{T}d_{1/2}d_{1/2}^{T}\left[\frac{\partial n_{1}}{\partial d_{1/2\left[j\right]}}n_{1}^{T}+n_{1}{\frac{\partial n_{1}}{\partial d_{1/2\left[j\right]}}}^{T}\right]d_{2}\;,$$

$$\frac{\partial c_{p12}}{\partial d_{2/1\left[j\right]}}=2d_{1}^{T}d_{1/2}d_{1/2}^{T}\left[\frac{\partial n_{1}}{\partial d_{2/1\left[j\right]}}n_{1}^{T}+n_{1}{\frac{\partial n_{1}}{\partial d_{2/1\left[j\right]}}}^{T}\right]d_{2}\;,$$

and

$$\frac{\partial c_{p12}}{\partial d_{2\left[j\right]}}=d_{1}^{T}d_{1/2}d_{1/2}^{T}R\left(\theta_{1},n_{1}\right)e_{\left[j\right]}\;.$$
